# Voltage Gated Sodium Channel Genes in Epilepsy: Mutations, Functional Studies, and Treatment Dimensions

**DOI:** 10.3389/fneur.2021.600050

**Published:** 2021-03-24

**Authors:** Ibitayo Abigail Ademuwagun, Solomon Oladapo Rotimi, Steffen Syrbe, Yvonne Ukamaka Ajamma, Ezekiel Adebiyi

**Affiliations:** ^1^Covenant University Bioinformatics Research, Covenant University, Ota, Nigeria; ^2^Department of Biochemistry, Covenant University, Ota, Nigeria; ^3^Clinic for Pediatric and Adolescent Medicine, Heidelberg University, Heidelberg, Germany; ^4^Department of Computer and Information Sciences, Covenant University, Ota, Nigeria; ^5^Division of Applied Bioinformatics, German Cancer Research Center (DKFZ), Heidelberg, Germany

**Keywords:** seizures, gain-of-functions, loss-of-functions, depolarization, VGSC

## Abstract

Genetic epilepsy occurs as a result of mutations in either a single gene or an interplay of different genes. These mutations have been detected in ion channel and non-ion channel genes. A noteworthy class of ion channel genes are the voltage gated sodium channels (VGSCs) that play key roles in the depolarization phase of action potentials in neurons. Of huge significance are *SCN1A, SCN1B, SCN2A, SCN3A*, and *SCN8A* genes that are highly expressed in the brain. Genomic studies have revealed inherited and *de novo* mutations in sodium channels that are linked to different forms of epilepsies. Due to the high frequency of sodium channel mutations in epilepsy, this review discusses the pathogenic mutations in the sodium channel genes that lead to epilepsy. In addition, it explores the functional studies on some known mutations and the clinical significance of VGSC mutations in the medical management of epilepsy. The understanding of these channel mutations may serve as a strong guide in making effective treatment decisions in patient management.

## Introduction

Epilepsy is a neurological disorder with spontaneous and re-occurring seizures that are borne from a population of cortical neurons, exhibiting hyper-synchronized discharge of high current action potentials in the brain (1, 2). Etiologically, epilepsy may have genetic, infectious, metabolic, structural, immunological, or unknown origin (3). Structural epilepsies are acquired following brain trauma, stroke, injuries, inflammations or infections, unlike genetic (formerly idiopathic) epilepsies which are primarily due to underlying genetic mutations (3). Studies in this field have shown that epilepsy may have a combination of two or more etiologies (4). For instance, many disorders involving the central nervous system (CNS) malformations such as tubulinopathies, tuberous sclerosis and polymicrogyria are comorbid with epilepsy and have genetic causes (5). The discovery of several ion channel gene mutations in epilepsy has led to categorizing epilepsy as a channelopathy. Epilepsy associated channel proteins fall in the class of ionotropic ligand receptors for major neurotransmitters (such as acetylcholine {muscarinic} receptors, glutamatergic receptors and GABAergic receptors) and the voltage gated ion channel proteins including sodium (Na^+^), calcium (Ca^2+^), potassium (K^+^), and chloride (Cl^−^) channels (6) are involved in bio-signaling in the CNS.

Voltage gated sodium ion channels (VGSCs) are heteromeric proteins involved in the generation and propagation of action potentials in brain neural cells (7). They are membrane-associated proteins and are responsible for conducting currents of sodium ions down their concentration gradient into the cell (8). Mutations associated with these channel genes are the most common cause of genetic epilepsies, with thousands of them reported in diverse populations (9). To understand the role of genetic variants in epilepsy, many studies have focused on the possible functional implications of a vast array of VGSC mutations. Often, these studies involved attempts to understand the mechanism by which induced mutations alter neuron physiology, using model systems. Some functional studies involved heterologous expression of mutated channel proteins in human embryonic cell lines or human induced pluripotent stem cells (hiPSCs) and the study of the aberrant network conduction in these transfected cells using voltage patch clamp techniques. Other methods involve the use of genetically modified rodent models (10) [e.g., Q54 mice, bearing the 879GAL881QQQ mutation in *SCN2A* (11)]. Several strategies for functional studies have been developed, which has helped to unravel the underlying molecular mechanisms by which many mutations trigger seizures. These have exposed other knowledge areas like the impact of mutations in modifier genes and the understanding of differences in patients' response to drugs. Therefore, this review aims to discuss VGSCs mutations in the alpha and beta subunit genes of sodium channels that lead to epilepsy, some functional studies of these mutations and their significance to epilepsy management.

## Search Criteria

A review of recent relevant literature was done on PubMed and GoogleScholar. Advanced search was done using the following keyword combinations: *SCN1A* mutations in epilepsy, *SCN1B* mutations in epilepsy, *SCN2A* mutations in epilepsy, *SCN3A* mutations in epilepsy, *SCN8A* mutations in epilepsy and functional studies of VGSCs in epilepsy. Search results (articles) containing the following word combination were excluded: Anticonvulsant properties of plant extracts, bioorganic compounds synthesized as antiepileptic drugs (AEDs), mechanism of action of new AEDs, clinical trials of drugs against epilepsy, heterocyclic compounds, imidazoles sulfonamides with anticonvulsant properties, quantitative structure activity relationship (QSAR) models for VGSC functions, biomarker studies in epilepsy syndromes and clinical trials of AEDs.

## VGSC Genes and Their Tissue Distributions

There are nine different sodium channel α-subunit genes that code for Nav1.1 to Nav1.9 (12) channel proteins. *SCN1A* to *SCN5A* encode Nav1.1-Nav1.5 proteins, while *SCN8A* to SCN11A encode Nav1.6-Nav1.9 proteins, respectively (13). On the other hand, five different ß-subunit proteins have been reported, namely: ß1 (product of *SCN1B*), ß1B (product of *SCN1B* splice variant), ß2 (*SCN2B*), ß3 (*SCN3B*), and ß4 (*SCN4B*) (14). In humans, the expression of VGSCs follows a tissue dependent pattern and each sodium channel possesses cognate kinetic properties that are both native to the tissue and relate to its function. *SCN1A, SCN2A*, and *SCN3A* have their locus clustered within a 600 kb region on the long arm of the *Homo sapiens* chromosome 2q24. The following are the VGSC α subunit genes highly expressed in the human brain: *SCN1A, SCN2A, SCN3A*, and *SCN8A* (Table 1). Hence, these are the genes involved in epilepsy pathogenesis. Other sodium channels α subunit genes that have high expression in muscles are involved in other pathologies. For example, *SCN4A* mutants cause hypokalemic periodic paralysis (type 2) (15), myotonia congenita and myasthenic syndrome (16) while *SCN5A* variants result in atrial fibrillation (type 10) (17), brugada syndrome (type 1) (18), and cardiac death (19) (Table 2). As for ß subunit genes, they display a wide distribution pattern in the brain. The proteins encoded by *SCN1B* (i.e., β1 and β1B are developmentally regulated). The β subunits occur as transmembrane polypeptides having their amino terminus protruding extracellularly and their carboxyl terminus protruding intracellularly, interacting with the proteins of the cytoskeleton. A conserved immunoglobulin (Ig) region is located at the extracellular portion, just like cell adhesion proteins (37). These proteins are found in close interactions with cellular adhesion proteins (38). In fact, some studies have shown the adhesive property of β subunits in the brain cells (39). Apart from seizure disorders, *SCN1B* variants also cause artrial fibrillation (type 13) and brugada syndrome (type 5) (27, 30).

**Table 1:** Sodium channel genes involved in epilepsy, proteins encoded, genetic loci, and distribution in human tissues.

**Sodium channel gene**	**Protein encode**	**Chromosomal loci**	**Major tissues expressed**	**Major developmental stage**
*SCN1A*	Nav1.1	2q24.3	Major sodium channel in inhibitory interneurons of the brain and spinal cord. Also expressed in lungs and testes.	Neonates, infant and young children
*SCN1B*	Na1.1b	19q13.11	Axon initial segment (AIS) of Inhibitory neurons of brain and heart.	Infant and young children. β1B predominates in embryonic brain
*SCN2A*	Nav1.2	2q24.3	AIS and distal portions of principal excitatory neurons of the brain. Also expressed in kidneys.	Prenatal and infant brain, low expression on adult brain
*SCN3A*	Nav1.3	2q24.3	AIS of principal excitatory neurons of brain and adrenal tissues.	Prenatal stage, Neonates, infants, and adults
*SCN8A*	Nav1.6	12q13.13	Nodes of ranvier, AIS and distal regions of both excitatory and inhibitory neurons of the brain cortex and cerebellum.	Low in neonates, Prevalent in adults

**Table 2:** Other pathologies associated with sodium channels.

**Genes**	**Pathological conditions**	**Pathology description**	**Inheritance pattern**	**References**
*SCN1A*	Familial hemiplegic migraine-3 (FHM3)	This disorder involves severe headache triggered by sensory disturbances like flash of light, noise, etc. In FHM3, the migraine occurs with intense weakness of half of the body.	Autosomal dominant	(20)
*SCN2A*	Episodic ataxia, type 9	A disorder characterized my poor muscular coordination, pain, dizziness, sluggishness, poor speech, and difficulty with movement of the limbs	Autosomal dominant	(21, 22)
*SCN4A*	Hyperkalemic periodic paralysis, type 2	A pathologic condition involving intense bilateral weakness and loss of muscle tone as a result of elevated levels of serum potassium	Autosomal dominant	(23, 24)
	Paramyotonia congenital	Individuals with this disorder experience skeletal muscle rigidity due to inability of the myocytes to relax appropriately after contraction. It is often induced by cold or exercise and begins at an early age.	Autosomal dominant	(24)
	Congenital Myasthenic Syndrome 16	A disorder characterized with severe muscle weakness, bulbar palsy, developmental delay and respiratory issues. It arises due to aberrant signal transmission at junctions where neurons interact with muscles.	Autosomal recessive	(16, 25)
	Hypokalemic periodic paralysis, type 2	A pathologic condition involving intense bilateral weakness as a result of reduced levels of serum potassium.	Autosomal dominant	(15, 26)
*SCN5A*	Brugada syndrome (type 1) *SCN1B* causes Brugada syndrome (type 5)	This disorder causes unexpected death, often in adults. It is caused by irregular contraction of the heart ventricules	Autosomal dominant	(18, 27)
	Sick sinus syndrome 1	A condition that occurs both in the elderly and in infants (congenitally). It is characterized by very slow heart beats, fainting and fatigue.	Autosomal recessive	(28)
	Long QT syndrome 3	A condition marked by arrthmia, seizures, and/or fainting. Physiologically, it occurs when repolarisation of the heat myocytes is dysfunctional, resulting in excessively fast heart beats. A common trigger is exercise.	Autosomal dominant	(18)
	Sudden infant death syndrome	A condition characterized by sponstaneous, unexplained death of a child during their 1st year of life, especially during sleep.	Autosomal recessive	(19)
	Cardiomyopathy, dilated with conduction disorder, type 1E	A progressive disorder of the heart characterized by heart enlargement, shrt breath, and failure to pump blood effectively.	Autosomal dominant	(29)
	Atrial fibrillation (type 10) *SCN1B* causes Atrial fibrillation (type 13)	This disorder is more prevalent in older adults. It is marked by atrial tarchycardia and arrhythmia.	Autosomal dominant	(17, 30)
*SCN8A*	Cognitive impairment with or without cerebellar ataxia	Children with this disorder manifest intellectual delay and developmental delay.	Autosomal dominant	(31)
*SCN9A*	Primary Erythermalgia	This condition is marked by inflammation of the extremeties (hands and feet), with burning sensations, pain, swelling and redness after exposure to stress or exercise. It may begin from infancy and progress till adulthood. It is a peripheral nervous system disorder.	Autosomal dominant	(32)
	Congenital insensitivity to pain and Hereditary, Sensory and Autonomic Neuropathy(HSAN), Type IID	Persons with this condition suffer from hereditary sensory and autonomic disorder that makes them unable to sense pain and heat/cold sensations. It begins from infancy and affects other autonomic functions.	Autosomal recessive	(33, 34)
	Paroxysmal extreme pain disorder (PEPD)	PEPD is associated with painful stooling or urination and flushing of the skin. The disorder is inherited.	Autosomal dominant	(35)
	Small fiber neuropathy and neuropathic pain	Adults with this condition suffer extreme pain and or itchiness in their extremities (hands and feet), due to certain triggers like heat. It's a disorder that affects the peripheral nerves.	Autosomal dominant	(36)

*SCN2A, SCN3A*, and *SCN8A* are three main α subunit genes that are mostly expressed on excitatory neurons. On inhibitory interneurons, *SCN1A* has high expression while *SCN8A* is also expressed to lesser extent (40, 41). The expression of these channels is usually concentrated on the AIS of neurons. However, *SCN2A* are mildly expressed on the soma and dendrites since they may promote back propagation of action potentials to the cell body (42). Nav1.6 is present mainly in the nodes of ranvier and distal portions of the AIS, but shows lower distribution on the dendrites and soma (43). Nav1.6 plays a higher role in action potential generation than Nav1.2 because of its relatively lower threshold for activation. Both channels differ in their functions despite the strong similarities they exhibit. Due to the differential expression patterns of sodium channels on inhibitory and excitatory neurons, polymorphisms in them tend to result in different seizure patterns and syndromes.

## *SCN1A*: Role in Epilepsy and Epilepsy Management

*SCN1A* mutations are the most prevalent amongst all VGSCs mutations in epilepsy and over 1,250 pathogenic variants are responsible for various epilepsies (44). In fact, amongst all epilepsy genes, *SCN1A* mutations are the most implicated (6, 45). Most Dravet syndrome (DS) and Generalized Epilepsy with Febrile Seizures Plus (GEFS+) cases have mutations in *SCN1A*. Both inherited and *de novo* mutations in *SCN1A* genes cause epilepsy and epileptic encephalopathies. For example, over three-fourth of DS cases are due to *de novo* mutations in *SCN1A* genes, while *SCN1A* mutations causing GEFS+ are frequently inherited (46). Many *SCN1A* mutations cause loss-of-function [e.g., *de novo SCN1A* mutations leading to DS occur as loss-of-function mutations on inhibitory interneurons (6)]. These mutations decrease the activity of GABAergic inhibitory interneurons (6). *SCN1A* variants in seizure disorders may occur as various mutation types such as missense, nonsense, protein-truncating variants, etc. For instance, about 50% of all DS cases arise from missense mutation while many others arise from non-sense mutations, deletions, frameshifts and splice-site variants (47, 48). Although DS and GEFS+ can be used as prototypes to study *SCN1A* variants, these mutations are also involved in other sub-types of epilepsy (13, 49).

Initially, research done using heterologous expression systems proposed that *SCN1A* mutations cause gain-of-function arising from dysfunctional inactivation of channel (50). Later, this suggestion was challenged after a host of loss-of-function mutations were discovered in patients with GEFS+. The effect of the deleterious missense mutations often results in a change in the biophysical properties of the Nav1.1 channel, causing a positive shift in the voltage dependence of activation with or without very slow inactivation of channel (51). Using bacterial artificial chromosome (BAC) transgenic mouse model, Tang et al. (52) reported that the effect of the R1648 mutation of *SCN1A* channel was a distorted resilience from inactivation, thereby implying loss-of-function. More recently, functional studies affirming loss-of-function were reported by Kluckova et al. (53) who investigated nine known and five novel variants, and observed clear partial and total loss-of-functions in different *SCN1A* variants. Four of them, E78K, E1587K, W384X, and R1596C resulted in mutant channels that failed to produce any measurable sodium currents (total function loss) while two, E788K and M909K resulted in partial loss. Three other variants (D249E, E78D, and T1934I) prevented the channel from recovering from the active state as required. Since *SCN1A* channels are majorly expressed on inhibitory interneurons, such loss-of-function mutations are expected to result in hyperexcitability.

Seizure manifestations and neuro-behavioral alterations observed in different epilepsies depend on the nature of mutations identified in sodium channels. Hence, VGSC gene screening is vital in epilepsy management. The total loss-of-function mutations in *SCN1A* results in more severe disorders. Meanwhile, the amino-acid-substituting point mutations causing change in function of sodium channels contribute to less severe epileptic disorders (6). This is exemplified in DS that arises from the loss-of-function mutation in *SCN1A* gene which is a more severe epileptic disorder than the GEFS+ (6, 9, 54). Zhang et al. (55) showed the relationship between clinical presentations of 13 children with SMEI having multiple seizure types and the incidence of having *SCN1A* mutations. Out of the 13 cases examined, 10 individuals with mutations in *SCN1A* were identified. They include 1 frameshift and 9 non-synonymous (seven missense and two non-sense) mutations. The authors concluded that despite identifying a well-known electroclinical pattern in these patients, a definitive genetic diagnostic approach involving *SCN1A* screening must be carried out for proper patient management. In another study involving 59 Italians and one Spanish patient having cryptogenic epilepsies, Zucca et al. (56) reported 12 *SCN1A* mutations. The mutational screening of *SCN1A* in 13 infants showed the presence of missense, non-sense, and frameshift mutations in patients with DS. Only 15% of the patients had abnormal brain imaging results, while most of them had normal electroencephalographic (EEG) activity (55). The consideration for genetic diagnosis in patients with epilepsy should not be teased out despite the absence of definite EEG results.

Microduplications in the 2q24.3 chromosomal region spanning through the genetic loci for *SCN1A, SCN2A*, and *SCN3A* that might influence expression levels of *SCN2A* were reported (57). The screened family seemed to manifest a seizure pattern that was different from the typical *KCNQ2* or *SCN2A* associated syndromes. *SCN1A* mutations showed lesser degree of electrophysiological alterations than *SCN3A* mutations. Hence, more intense phenotypes were observed in the former (58). Also, frameshift mutations have been identified as one of the major underlying mutations in *SCN1A* responsible for DS (59). Tuncer et al. (59) reported a new *SCN1A* mutation in a 10-year-old Turkish boy who had seizure onset at 8 months old. Mutational screening of *SCN1A* revealed the presence of a deletion c.4018delC that resulted in a shift in the open reading frame, causing the premature introduction of a stop codon. The patient had a history of refractory seizures, which could not be effectively managed using monotherapy of phenobarbital, valproic acid, or other AEDs. Upon genetic screening, patient management took the course of a combined therapy using valproic acid (VPA) and stiripentol that proved effective. The authors reported the presence of a borderline phenotype rather than the regular DS (59). Genetic investigation improves diagnosis and may help in selecting better effective combination therapies in epilepsy management.

Studies by Shi et al. (60) showed the association between single nucleotide polymorphisms (SNPs) in *SCN1A* in epileptic Chinese patients and their recovery rates when valproic acid was administered. A common *SCN1A*allele, rs3812718G>A, was linked with a positive drug response to valproic acid in the studied Chinese population (60). This was inconsistent with reports from previous studies (61) suggesting the discontinuation of sodium channel blockers as effective therapy against seizures in *SCN1A-*positive patients. This indicates that some population-based variants may alter patients' response to drugs, but this may be absent in other populations.

Fang et al. (62) reported a study of 24 epileptic patients of Chinese origin who had previously tested positive on *SCN1A* mutation screening. About 67% of the patients had missense mutations and about 17% had non-sense mutations. One patient had splice-site and frameshift mutations while large deletions were identified in only two of the patients. The patients' genetic background with respect to *SCN1A* mutations had impact on the medications that proved effective. For instance, ~33% of the patients were refractory to treatment with antiepileptic drugs (AEDs) while ~46% were drug responsive. The dietary intervention using ketogenic diet proved successful in three patients, who were rather non-responsive to AEDs. The use of sodium channel blockers proved unsuccessful in over 41% of the patients (62). Sodium channel blockers are being discontinued as a choice of treatment for the management of *SCN1A*-related seizure disorders due to their proven exacerbating effects in the presence of such mutations. In epilepsy management, the importance of mutational screening cannot be overemphasized because it gives a road map to effective diagnosis and treatment, while avoiding therapies that may aggravate the condition.

The discovery of pathogenic haploinsufficiency in DS has contributed to the clinical management of patients. Sodium channel antagonists like lamotrigine, phenytoin and carbamazepine are being discontinued in patients with DS and other epilepsy syndromes associated with *SCN1A* mutations (61). This was not the case with earlier studies on the management of *SCN1A* disorders. For instance, Krikova et al. (63) studied the effective therapeutic dose of lamotrigine (a sodium channel antagonist) in heterozygous and homozygous *SCN1A* mutations and observed the highest effective dose amongst those with homozygous *SCN1A* mutation. Today, the application of adjunctive therapies is highly sought after. However, their efficacy in the absence of adverse drug response is a matter of concern. An example is Cannabidiol (CBD) oil which has resulted in effective seizure management in patients with DS but several adverse events ranging from moderate to severe have been observed (64). New AEDs are already being considered for the treatment of DS. Two important drugs amongst these are stiripentol and fenfluramine. Although it originated in the 1970's, stiripentol is increasingly gaining attention for its minimal adverse reactions and its capacity to effectively manage seizures in DS, epilepsy of infancy with migrating focal seizures (EIMFS) and other intractable seizures (65). The initial uproar regarding the safety of humans using fenfluramine in weight management has gradually reduced with the release of new findings showing its efficacy in seizure management for patients with DS. Ceulemans et al. (66) recorded 70% success in a study involving the treatment of patients with DS with fenfluramine. On an average, the patients stayed seizure-free for 6 years. The result of a 5-year follow-up of the same cohort, revealed the safety of fenfluramine in patients with DS with effective seizure management, and only two patients recorded cardiovascular thickening (67). Another study involving nine patients revealed that over 70% of patients on fenfluramine had reduction in motor seizures (68, 69). These studies applied fenfluramine at low doses. Lagae et al. (70) reported the outcome of a Phase III clinical trial which suggests that the drug is safe for use as a combination drug at low doses (<30 mg per day) for an extended treatment period over several years with no adverse cardiovascular effects in young patients. These results suggest a breakthrough for the treatment of *SCN1A*-seizure disorders, with DS as a prototype. More clinical trials for fenfluramine are still ongoing and interesting findings are being reported.

Using a combination of immunochemistry and immunofluorescence, Guo et al. (71) reported an increased level of expression of sodium channel alpha subunits 1 and 3, and β1 subunits in the hippocampal region of spontaneous epileptic rats. This study revealed that cellular changes that result in the overexpression of some channel proteins may have critical roles to play in seizure generation. Also, the impact of epigenetic modifications is still gaining attention in epilepsy studies. Drugs that can specifically inhibit the activities of DNA methyl transferases (DNMTs) and Histone deacetylases (HDACs) are already being applied, especially in patients having temporal lobe epilepsy (TLE) (72). Therefore, proper understanding of epigenetic mechanisms of epileptogenesis would lead to the development of more adjunctive therapies in the management of epilepsy.

Functional studies (Table 3) can help detect gain or loss-of-function variants that may guide drug design and understanding of the relationships between variants and drug response. An example of the impact of genetic variant on drug response was recently reported by Zhao et al. (77) who examined the association between the *SCN1A* SNPs; rs3812718 and rs2298771 and the response to carbamazepine in a Chinese population. Resistance to drugs was observed with rs3812718 but not with rs2298771.

**Table 3:** Regions of SCN1A with functionally validated epilepsy variants with references.

**Gene**	**Amino acids affected by missense variants**	**Deletion regions**	**Protein-truncating variant regions**	**References**
***SCN1A***	M145,G177,D188,I227,L263,N301,Y325, R393,Y426, E788,T808,R859,R865,T875,F902, M909,H939,R946,C959,G979,V983,N985,L986,N1011,T1174,W1204,K1270,V1353,F1415, Q1489,R1575, E1587, R1596,V1611,P1632,R1648,L1649,I1656,R1657,F1661,G1674,A1685,T1709,G1749,F1765,F1808,F1831,M1841, M1852,D1866,T1909, R1916, Q1923, R1927, T1934	V806-L863,F1289,T160-Y202,V1335-V1428,V806-L863	E78, Y159,R222,W384,R712,R1234, R1245,R1396, R1892,R1407, R1645	(44, 48, 52, 53, 73–76) and *SCN1A* database (https://scn1a.caae.org.cn/functional_studies_eletrophy.php)

*SCN1A database provides detailed information on functional studies of SCN1A mutations. Above, we have included references not yet updated on the website*.

Progress is being recorded in functional studies of channel mutations. Liu et al. (48) was the first to apply neurons derived from human induced pluripotent skin fibroblasts to study functional effects of *SCN1A* mutation in a neuron-related disorder. Studies of splice-site mutation of *SCN1A* revealed clear loss-of-function using induced iPSCs. The authors compared results of using heterologous expression systems like tsA201 and xenopus oocyte cells with results of patient- and control-derived pyramidal and bipolar neurons studies. While transfected heterologous system had little or no detectable current, the hiPSC-neurons delivered hyperexcitation as in the case with typical DS (48). They observed that hiPSCs neurons utilized the actual genetic architecture of the patients' neurons to study functional impacts rather than using other mammalian cells that may introduce some bias into the results due to inter-specie differences. Also, hiPSCs permit the studying of mutations in the presence of the patient's own genetic modifiers hereby providing more effective tailor-made therapies. Furthermore, neurons are more suitable for expressing channel biophysical properties than other mammalian cells and they serve as better models. Xie et al. (44) recently demonstrated the high potential of using hiPSC-neurons for functional studies by applying the K1270 *SCN1A* mutation. They observed a reduced depolarization signal in the mutant channel which suggested loss-of-function. Likewise, they realized that this approach improved the understanding of molecular mechanism by which mutations exert their effects on neurons.

Genetic screening may reveal population-based variants responsible for differences in response to drugs in diverse populations. One advantage of identifying population-related variants with their functional studies is that it will pave way for the development or determination of drugs that are more suitable for that population.

**Figure 1:**
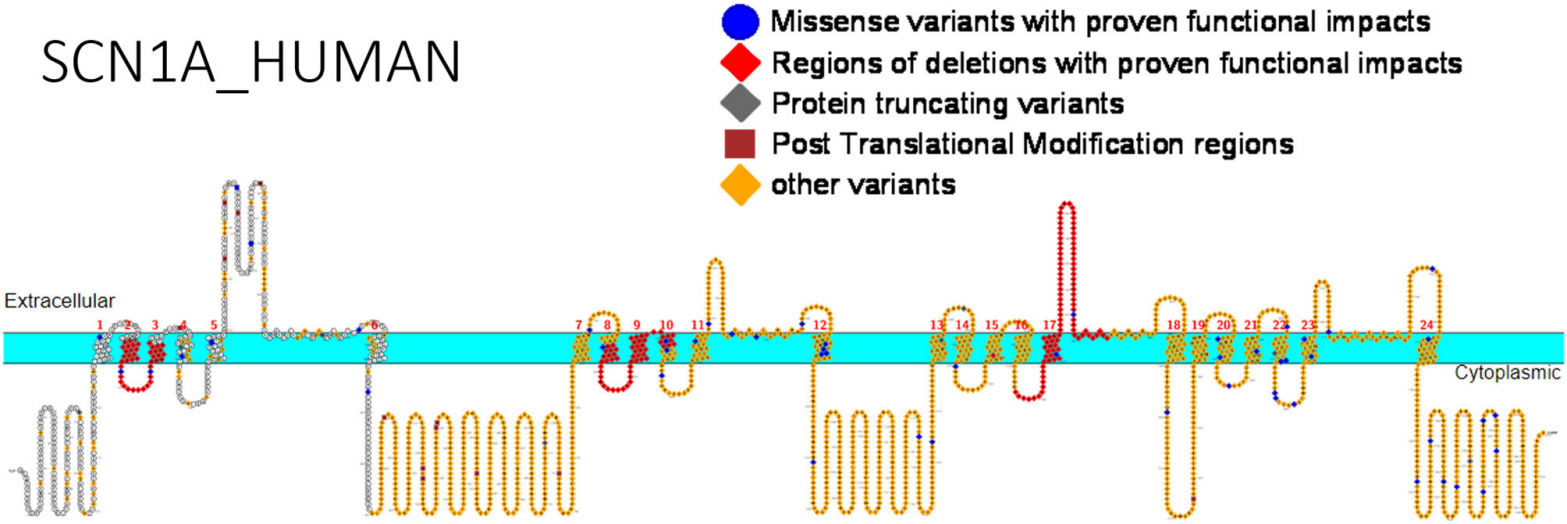
Primary structure of *SCN1A* protein showing regions with functionally validated epilepsy-variants from literature. Protein-truncating variant region (black), regions of deletions with proven functional impacts (red), regions of missense variants with proven functional impacts (blue), regions of post-translational modifications (brown), other variants (orange). [Created using Protter (78). Visualization link: https://bit.ly/2NWfncp].

We have collated the *SCN1A* regions having variants whose functional studies have been reported in Figure 1, while Table 3 shows the affected regions with references.

## *SCN1B*: Role in Epilepsy and Epilepsy Management

Mutations in the modulating ß subunits of the type 1 sodium channel genes have equally been confirmed as another cause of epilepsy. Wallace et al. (79) was the first to report a pathogenic missense mutation, p.(C121W), in *SCN1B* gene inherited in an autosomal dominant pattern in an Australian GEFS+ patient. Studies using heterologous expression systems revealed that this mutation hindered the function of β subunits both as an ancillary protein and adhesive protein in addition to altering gating characteristics of the channel. It also disrupts the Ig loop of the subunit, hereby inhibiting glycosylation (38, 80–82). Many mutations have been identified in this gene since then. One homozygous mutation was reported in a case of epileptic encephalopathy (83). Another study identified four mutations in *SCN1B* having linkage to epileptic seizures. All these mutations were found on the extracellular portion of the protein (84). This portion on the modulating ß-subunit was essential in channel gating. The four mutations were identified in patients having GEFS+. The most occurring missense substitution was the point mutation replacing cysteine with tryptophan p.(C121W) (84). More insights into the roles of sodium channel β subunits have shown that they do more than simply modify the functions of the α-subunits. In the absence of α subunits, they can function as indicator molecules in electrically activated cells. In addition, they play key roles in brain morphogenesis, modification of potassium channels, regulation of channel expression and cell to cell interactions (10).

Mutations in *SCN1B* have been reported in patients having GEFS+ phenotype, DS and Temporal lobe epilepsy (TLE) (79, 84, 85). However, many of these mutations are localized in the extracellular region of the auxiliary channel protein. Most functional studies on *SCN1B* revealed loss-of-function mutations, especially interfering with their ability to augment VGSC gating (79). The functional implication of these β subunit mutations is that they may impede the role of the principal pore forming α-subunits (86). Meanwhile, molecular studies to unravel definite pathogenicity of mutations in *SCN1B* are still incomplete.

Kruger et al. (87) reported the functional studies of the missense mutation, p.(C121W) that was initially reported by Wallace et al. (79). Using an experimental model of childhood febrile seizures, the authors compared the response of heterozygous *SCN1B*-C121W (*SCN1B*+/w) mice with *SCN1B*-null allele. The *SCN1B*+/w mice exhibited higher predisposition to heat-induced paroxysms, suggesting a gain-of-function mechanism (87). The results were contrary to the initial conclusions from co-expression studies using *Xenopus* oocytes and mammalian cell lines that suggested a loss-of-function of channel protein as a mechanism of action for this mutation (38, 79, 80). The response of the channels to the sodium ion channel blocker; phenytoin, decreased in the presence of the *SCN1B* mutation p.(C121W) (88).

Patino et al. (89) examined the functional implications of the mutation, p.G257R, earlier identified by Patino et al. (83) in β1B subunit which is mainly expressed in embryonic brains. They demonstrated that unlike β1, the splice-variant, β1B, does not function as a transmembrane polypeptide. Rather, it served as a secreted protein acting as a ligand in neuronal growth. Furthermore, they revealed that the mutation might alter sodium ion currents in the brain, using Chinese Hamster Ovary cells co-expressing Nav1.3 and β1B subunits. Similarly, their results suggested that p.(G257R) mutation acted in a similar manner to a previously reported missense mutation p.(R125C) which inhibited the release of β1B polypeptide into the extracellular environment, hence annulling its function in neuronite outgrowth (89).

**Figure 2:**
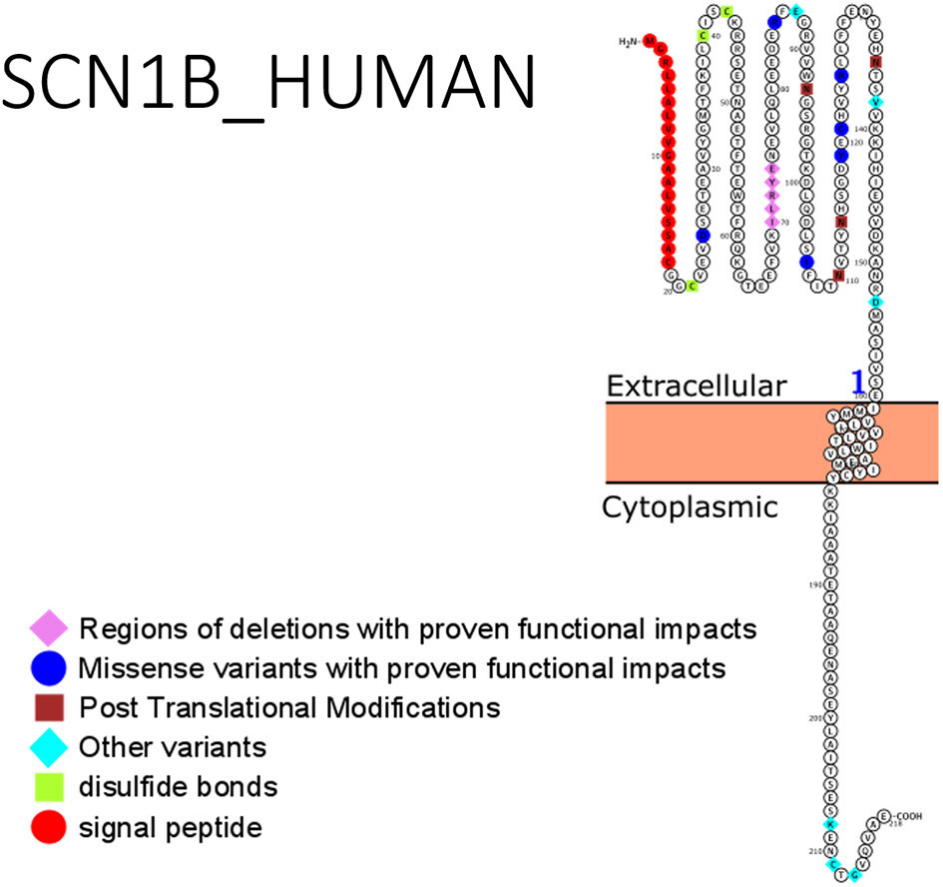
Primary structure of *SCN1B* protein showing regions with functionally validated epilepsy-variants from literature. Region of signal peptide (red), missense variant regions with proven functional impacts (blue), post-translational modifications (brown), other variants (aqua), regions of disulfide bonds (Lemon). [Created using Protter (78). Visualization link: https://bit.ly/366Lz6C].

Studies involving the mutant *SCN1B* gene in heterologous expression systems require co-expression with the α-subunit gene. Most mutations in *SCN1B* are found on the extracellular immunoglobulin fold (whose primary role is in cell to cell adhesion and interaction with the α-subunits) with few exceptions (e.g., one mutation on ß1B subunit was identified in its C-terminal domain). Phenotypic expression of these mutations and functional characterization suggested a loss-of-function mechanism. Additionally, the ß1 subunit had interactions with some types of potassium channels, thus aiding repolarization of these channels (90), by accelerating the activation of the potassium channel and suppressing its inactivation. In GEFS+ patients, where variants p.(C121W) and p.(R85C) of *SCN1B* have been described, there was a loss of effects of ß1 function on the Kv1.3 potassium channel (91). Identification and characterization of the roles of ß1 and ß1B variants in other forms of epilepsy, especially their effects on channel gating are of crucial importance. The role of *SCN1B* was recently demonstrated in developmental and epileptic encephalopathy. The mutation, R85C of *SCN1B* was identified as a loss-of-function variant (92). This was verified by Dang et al. (93) who studied the missense mutations: p.(R85C), p.(R85H), and p.(R89H). Dang et al. (93) confirmed a loss-of-function mechanism for the R85 variants. Clinical management of a child with refractory seizures proved ineffective with several combination therapies including levetiracetam, clobazam, Valproate and topiramate. However, the administration of Phenytoin resulted in effective management of the seizures (93). Despite this finding, the treatment of *SCN1B*-related epilepsy with sodium channel blockers remains inconclusive. There is still sparse information on the successful treatment in *SCN1B*-related epilepsy. Hence, more work is required, including functional studies using induced hIPSCs. Figure 2 shows *SCN1B* primary structure with regions having seizure-prone variants that have been functionally validated from literature while Table 4 shows affected regions with references.

**Table 4:** Regions of *SCN1B* with functionally validated epilepsy variants with references.

**Gene**	**Amino acids affected by missense variants**	**Deletion regions**	**Protein-truncating variant regions**	**References**
*SCN1B*	D25, R85, R89, I106, Y119, R125, C121	I70-E74	-	(79, 83, 85, 87, 94–97)

## *SCN2A*: Role in Epilepsy and Epilepsy Management

There are two isoforms of *SCN2A*: one is expressed in infancy while the other is expressed in adulthood. The adult isoform has lower threshold for excitation than those of a neonate, thus, it is more excitable (10). Reid et al. (98) reported about 20 pathogenic variants of *SCN2A* leading to benign familiar neonatal infantile seizure (BFNIS) and GEFS+. The pathogenic mutations in this gene have been reported in several epilepsies of different severities. There is an age dependent seizure resolution in BFNIS that relates to the physiological reorganization of the axon initial segment during development. This rearrangement leads to the substitution of *SCN2A* with *SCN8A* in the distal regions of the AIS. Thus, reducing the impact of the mutant *SCN2A* on neuronal function (99). *SCN2A* and *SCN8A* mutations have been reported in some patients with severe epileptic encephalopathies (100). Ogiwara et al. (101) reported another more severe epileptic encephalopathy arising from *de novo SCN2A* mutations with clinical manifestations that resemble those of DS. Whole exome sequencing is continuously having more relevance in epilepsy genetics, especially in its potential to reveal genes whose mutations may have epistatic interactions by acting as genetic modifiers of disease. Studies by Shi et al. (102) to examine the impacts of *SCN2A, GABRG2*, and *SCN1A* in DS amongst Japanese population showed 29 mutations in *SCN1A* and 3 in *SCN2A*. The missense mutations, F328V and D322N were inherited while R1312T was *de novo* (102). Many *SCN2A* mutations leading to refractory epilepsies are often *de novo* rather than inherited (103).

Frameshift mutations have been noted in *SCN2A* in children having intellectual disability (ID) without seizure history (104). The first non-sense, protein-truncating mutation p.(R102X) in *SCN2A* was reported in patients having refractory seizure and ID by Kamiya et al. (105). This mutation was heterozygous and absent in parents of the reported patients. Electrophysiologic analysis using HEK 293 cells and voltage patch clamp studies showed that the mutation caused a hyperpolarization shift. Liao et al. (99) examined the clinical presentations in one child with neonatal seizures accompanied by pain and episodic ataxia. The authors reported a missense mutation p.(A263V) whose functional consequence is a rise in sodium currents in the mutant channel (99). Despite these promising results, mutation studies of *SCN2A* in patients with seizure disorders have not shown clearly defined phenotypes unlike *SCN1A* and *SCN8A* (106).

The role of *SCN2A* mutations in Early Onset Epileptic Encephalopathy (EOEE) was reported by Nakamura et al. (107) in a study involving 328 individuals with various types of EE. Fifteen novel amino acid replacing mutations were reported with ~86% (12) occurring *de novo*. Amongst the cases studied, all mutations in patients having Ohtahara syndrome occurred in the polypeptide loop that connects two transmembrane regions of Nav1.2. A country-wide study of patients with acute encephalopathy and fever-induced status epilepticus was recorded. Two amino acid substituting mutations in *SCN2A* were identified, one was previously identified in patients with DS p.(F328V) and the other was novel p.(I172V) (108).

Missense mutations in *SCN2A* have shown varying effects on the mutant protein. Cell culture studies using two BFNIS gene mutations revealed a depolarizing shift in voltage dependence of inactivation and an elevated/persistent current generation depicting gain-of-function as one mechanism in which *SCN2A* mutations cause excitability (109). Gain-of-function mutations in Nav1.2 cause seizures since these sodium channels are highly expressed on excitatory neurons. In a study conducted by Xu, Thomas (110) it was evident that gain-of-function mutations of the neonatal Nav1.2 predisposes to a higher seizure frequency than those of adult. The mutation in neonatal Nav1.2 increased excitability to a level that was similar to that expressed by the adult phenotype (110). This explains the self-limiting effect of BFNIS with advancement in age (10). Further studies are required to identify and characterize more *SCN2A* mutations and their involvements in epilepsies.

*SCN2A* mutations have been shown to be the second most prevalent mutation following *KCNT1* (111) in patients having EIMFS. Treatment with phenytoin at high dosage proved effective in 75% of the cases having multiple epileptic syndromes including EIMFS and OS (112). The authors reported 11 missense mutations in *SCN2A* including a deletion mutation, that resulted in an amino acid substitution (112). Reports from Matalon et al. (113) supported the claim that *SCN2A* mutations are involved in early infantile epilepsies. They presented three children having refractory seizures with *de novo SCN2A* mutations. The three patients reported were found to be negative for mutational screening in the following epilepsy genes: *FOXG1, CDKL5, MECP2, ARX, SCN1A, STXBP1, SLC25A22, PCDH19, SPTAN1*, and *ARHGEF9*. The study showed that *SCN2A* does not merely act as a disease modifying gene but can independently trigger seizures. Three amino acid substituting *de novo* mutations were reported (113). Exome studies by Baasch et al. (103) revealed one new *de novo* missense mutation p.(R1882L) in *SCN2A* in patients with epilepsy and intellectual disability. The authors presented another supporting claim that pathogenic *SCN2A*mutationswere often *de novo*.

Zeng et al. (114) reported the clinical phenotypes of 21 children having mutations in *SCN2A*. Approximately 48% of the cases had inherited the mutations from their parents while the remaining had *de novo* mutations in *SCN2A*. Majority (about 86%) of the children had partial seizures and most of the patients (about 90%) manifested more than one seizure type. Less than one-third of the examined patients had infantile spasms. *De novo* mutations in this gene were found to be associated with delayed development.

Seizures arising from *SCN2A* mutations are well-controlled by high therapeutic doses of the sodium channel blocker, phenytoin (112). Two other sodium channel antagonistic seizure medications (lidocaine and mexiletine) were reported to be effective anecdotally in infants having intractable epilepsy due to *de novo* mutations in *SCN2A* (115). This was after a combination therapy including topiramate, levetiracetam, lacosamide, oxcarbazepine, and phenytoin proved abortive. Ketogenic diet including adjunctive therapies involving treatments with pyridoxine, folinic acid, pyridoxyl-5-phosphate was also applied to no avail. This medication was replicated in another patient carrying a *de novo SCN2A* missense mutation p.(G999L) and a successful seizure management was recorded (115). Conflicting reports in the management of *SCN2A*-related seizures were recorded until 2017 when Wolff et al. (116) identified a trend in the functional studies of *SCN2A*-variants. They reported that loss-of-function in *SCN2A* variants were more associated with seizures beginning over 3 months of age and were not properly managed using sodium channel antagonists. However, in infants <3 months a gain-of-function trend was observed and the administration of sodium channel blockers proved very effective (116). This trend requires further studies for clear validation and clinical applications.

As in the case with *SCN1A* mutations, dietary interventions are sometimes effective in managing *SCN2A*-related seizures. The use of modified Atkins diet in one Chinese child was found to reduce seizures that presented with refractory seizures, infantile spasms and delayed development including autism-related characteristics. An *SCN2A* mutation p.(E1211K) which had been earlier identified in an infantile spasm case was detected (117). In spite of past failed attempts in the use of ketogenic diets in managing *SCN2A*-seizures (115), ketogenic diet has proved successful in managing *SCN2A*-epilepsy when adopted early enough (118, 119).

**Figure 3:**
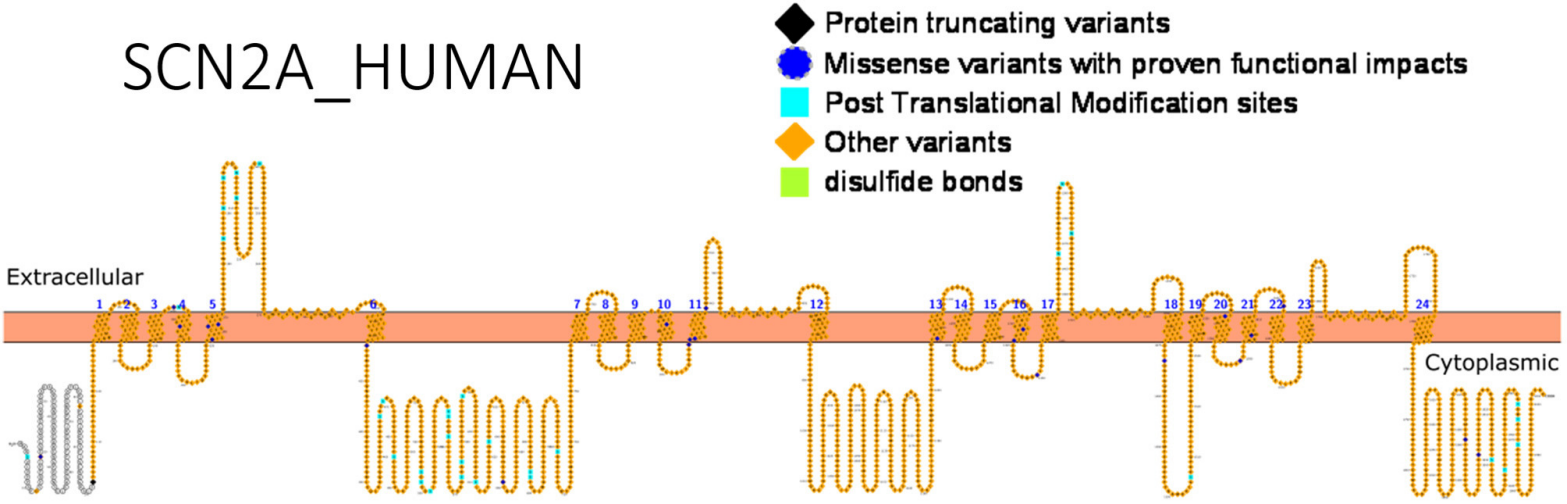
Primary structure of *SCN2A* protein showing regions with functionally validated epilepsy-variants from literature. Protein-truncating variant regions (black), region of missense variants with proven functional impacts (blue), post-translational modifications (aqua), other variants (orange) and regions of disulfide bonds (lemon) [Created using Protter (78). Visualization link: https://bit.ly/2HLNnbW].

Apart from determining the treatment route, genetic variants may also determine drug response, and this has been reported for *SCN2A*. An association between *SCN2A* variants and selective drug response to valproic acid (VPA) was recently reported by Shi et al. (60) in Chinese patients. The allele, rs2304016A>G was negatively associated with response to VPA. Epistatic interactions amongst genes may equally determine how patients respond to drugs. The impact of epistatic interactions affecting response to valproic acid was studied in Chinese Han population (120). The following *SCN2A* SNPs had epistatic interactions influencing VPA response: rs10197716 and rs2119068, rs10197716, and rs11889342. Furthermore, interactions between *SCN2A* (rs7598931) and UDP-Glucuronosyltransferase-2B7 gene (UGT2B7) (rs12233719) were reported (120). Functional studies can help detect gain- or loss-of-function variants that can guide drug design, identify high risk population-related variants that are important for genetic testing and understand relationships between variants and drug response. Figure 3 shows *SCN2A* primary structure with regions having variants have been functionally validated from literature while Table 5 shows the affected regions with references.

**Table 5:** Regions of *SCN2A* with functionally validated epilepsy variants with references.

**Gene**	**Amino acids affected by missense variants**	**Deletion regions**	**Protein-truncating variant regions**	**References**
*SCN2A*	R19, R1902, R1882, R853, L1563, R1312, Y1589, R1319, L1563, L1330, M252, V261, A263, I1473, E1211, D649, F1597, V423, G899, N1622, G879-L881, G211, R223	-	R102	(11, 99, 101, 109, 121, 122)

## *SCN3A*: Role in Epilepsy and Epilepsy Management

The *SCN3A* gene is highly expressed in brains of neonates and adults. It is similarly expressed both in principal excitatory and inhibitory interneurons. Point mutations in *SCN3A* genes have been reported in patients with focal epilepsies (58, 123). Holland et al. (12) presented the first *SCN3A* mutations in cryptogenic focal seizures. In managing the patient's seizures, previous treatments that involved the administration of sodium channel blockers (carbamazepine or oxcarbazepine) had been futile. After mutational screening, the authors reported 1 new missense mutation (K354Q) in the polypeptide link between S5-S6 of domain I, and 3 novel synonymous variants in *SCN3A*. Electrophysiological studies of the missense mutation using *SCN5A* gene show a rise in sodium ion currents causing defects in inactivation in excitatory neurons. Although this suggests that the mutation acts through a gain-of function mechanism, the use of an isoform rather than *SCN3A* itself, may introduce a bias in the accurate measurement of the effect of this mutation. The first mechanistic study of *SCN3A* mutation using transfected neurons of the hippocampus was reported in 2010. Estacion et al. (124) provided the functional studies of the p.(K354Q) mutation (earlier reported by Holland et al. in 2008) in Nav1.3 channels using hippocampal cells from rats. The authors validated that this mutation increased ionic currents across the channel and decreased the threshold for Na+, resulting in hyperexcitation. A more improved approach now involves the use of neurons derived from patients' fibroblasts to express mutant channels and better results are being recorded in terms of studying the channels' biophysical properties. Functional studies of even more *SCN3A* mutations have shown an elevated ramp current in mutant channels, further suggesting gain-of-function (123).

Apart from gain-of-functions mutations, loss-of-functions mutations are implicated in focal epilepsies. Chen et al. (58) reported an *SCN3A* mutation in some patients with GEFS+. The identified mutation was consequential in creating a depolarizing shift in voltage-dependent activation and inactivation of the sodium channel, including delayed recovery from slow inactivation. Hence resulting in channel inactivity. Furthermore, Lamar et al. (125) identified a novel mutation in *SCN3A* yielding the mutant Nav1.3 protein p.(L247P) which is associated with focal epilepsy in childhood and delayed development. The authors demonstrated the role of a novel *SCN3A* mutation p. (L247P) in seizure pathogenesis. They reported that loss-of-function mutation in *SCN3A*, reduced trafficking of channel proteins or down regulated expression may result in epilepsy. Despite this, more studies have shown a prevalence of gain-of-function mutations for *SCN3A*. Zaman et al. (126) studied some *SCN3A* missense variants including p. (I875T), p. (P1333L), p. (V1769A), p. (R1642C), and p. (L1799Q). The result of biophysical studies on channel mutation showed a prevalence of gain-of-function with higher amplitude of inactivation and a lowered threshold of activating the channel compared to the wildtype channel. This effect was impeded by the application of 2 specific sodium channel blockers (lacosamide and phenytoin). Thus, implying their efficacy in managing *SCN3A* disorders. Mixed results of gain and loss-of-function are recorded because *SCN3A* is known to be expressed also on inhibitory neurons and this may explain why loss-of-function in this gene may also result in seizures.

Microduplication mutations have likewise been implicated as epilepsy-related *SCN3A* mutations. Yoshitomi et al. (127) reported 3 patients manifesting focal seizures and infantile spasms having microduplication mutation on the 2q24.3 chromosomal loci. Eight additional duplications were reported by Thuresson et al. (128). The region duplicated was overlapped and the spanned range approximately between 0.05 and 7.63 Mb in size. This resulted in an extra *SCN2A* copy which caused benign familial infantile seizures (BFIS), but had no link with cognitive decline (128). Recently, the role of deletion mutations has been expounded. A causative *de novo* deletion mutations in the 2q24.3 region was reported in a patient with West syndrome who also manifested symptoms of autism. This deletion (~1.1 Mb) covers the region encoding Nav1.2 and Nav1.3 (129).

**Figure 4:**
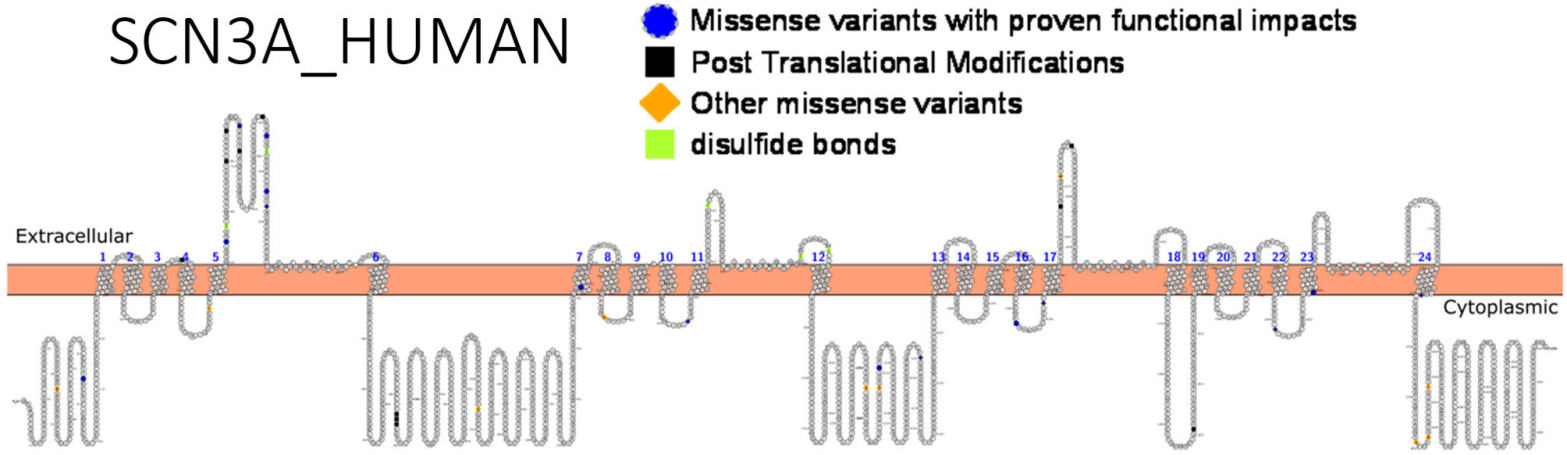
Primary structure of *SCN3A* protein showing regions with functionally validated epilepsy-variants from literature. Regions of missense variants with proven functional impacts (blue), regions of post-translational modifications (black), other missense variants (orange) [Created using Protter (78). Visualization link: https://bit.ly/2V8ZeDL].

Apart from *de novo* mutations, inherited mutations are known to cause *SCN3A-*seizure disorders. Using targeted exome sequencing including 412 epilepsy genes in 63 patients and their families, an inherited missense mutation p.(R621C) was identified in *SCN3A* but no functional studies was done (130). Zaman et al. (126) asserted that since many reported *SCN3A* variants occur *de novo*, they might have roles in modifying the effects of other pathogenic variants from different genes, which exhibit high penetrance. Functional analysis of these channel variants will assist in unraveling the molecular mechanism underlying phenotypic expression and shed more light on research to develop more effective therapies. Figure 4 shows *SCN3A* primary structure with regions having variants that have been functionally validated from literature while Table 6 shows the affected regions with references.

**Table 6:** Regions of *SCN3A* with functionally validated epilepsy variants with references.

**Gene**	**Amino acids affected by missense variants**	**Deletion regions**	**Protein-truncating variant regions**	**References**
*SCN3A*	I85, N302, R274, A343, K354, R357, I766, I875, D1111, V1323, P1333, R1642, N1657, V1769, E1160	-	-	(12, 124–126)

## *SCN8A*: Role in Epilepsy and Epilepsy Management

Mutations in *SCN8A* gene is the major underlying factor responsible for pathogenesis of *SCN8A* encephalopathies; a seizure disorder marked with early onset paroxysmal manifestations characterized by hypersynchronous electrical discharges originating in the brain. This usually occurs within the first 18 months after birth, particularly after the 3rd month (131). Clinical manifestations include cognitive disability and developmental delay. About half of the children involved did not walk or sit properly (or not at all), and often manifest defects with the peripheral limbic functions. One-tenth of them have experienced sudden unexpected death in epilepsy (SUDEP). The antiepileptic drugs in the class of sodium channel blockers often guarantee transient improvement in few of the affected patients. However, the control of the paroxysmal discharge is far from sufficient (132).

Recent works in genomics have identified *SCN8A* as an epilepsy gene. Nav1.6 are widely distributed on the AIS of principal excitatory neurons and inhibitory interneurons (133). Expression of Nav1.6 is not limited to the CNS but is largely expressed in PNS neural cells. The expression of *SCN8A* is highly concentrated at the AIS of both excitatory and inhibitory neurons (48). In both neuron types, Nav1.6 coordinates excitation of neurons (43, 134). Studies using *SCN8A* knockout mice revealed that this gene is primarily involved in neuronal excitability in excitable cells such as purkinje cells of the cerebellum, ganglion cells of the retina and hippocampal cells (135). Hence, these mice exhibit early (juvenile) death (136). Furthermore, Nav1.6 is the main VGSC at nodes of Ranvier of mature neurons (43), and its under-expression inhibited nerve conduction (137).

Initial genetic studies using rodents revealed that the *SCN8A* gene is also mutated in epilepsy with absence seizures, a genetic generalized epilepsy (GGE) (138). Papale et al. (138) was the first to demonstrate that *SCN8A* is also a candidate gene in epilepsy using a mouse model with absence epilepsy. Heterozygous *SCN8A*-knock out mice manifested unprovoked spike-wave discharges which is typical of absence epileptic seizures.

Early infantile epileptic encephalopathy Type 13 (also called *SCN8A* encephalopathy) is caused by mutations of the *SCN8A* gene encoding the alpha subunit of Nav1.6 channel protein (132). Family studies have repeatedly shown *de novo* mutation in affected individuals, often arising from gain-of-function missense non-synonymous mutations. The mutant sodium channel exhibit enhanced activity (139). In a report by Blanchard et al., the physiological change observed in a mutant Nav1.6 was dysfunctional channel inactivation and a hyperpolarizing shift in voltage dependence of excitation resulting in an untimely or early channel re-opening (140). This implies that such mutations are localized on the Nav1.6 channels located on the principal excitatory neurons.

Veeramah et al. (141) reported a case of *de novo* heterozygous missense mutation in a child with severe infantile encephalopathy which started at 6 months. It was characterized with epileptic convulsions during the 4th year and was associated with SUDEP at age 15. This was a p.(N1768D) mutation in the Nav1.6 sodium channel expressed in the brain. Upon the expression of this mutant gene in ND7 cells, an enhanced ramp current with a corresponding shift in quick inactivation that was in favor of depolarization was observed. This led to hyperexcitability in pyramidal neurons of the hippocampus. In addition to this, incomplete inactivation of the channel was detected and there was a depolarizing shift in the voltage dependence of inactivation of the mutant channel (141). Complete identification and characterization of this mutation was typical of a gain-of-function, associated with enhanced ramp current. Hence, they suggested that a gain-of-function mutation in *SCN8A* can cause epileptic encephalopathy. The implication of this new mutation was a dysfunctional channel with an impaired inactivation potential leading to neuronal hyperactivity. Several large exome sequencing projects have equally identified 11 *de novo* mutations of *SCN8A* in singleton patients with epilepsy with or without intellectual disability (135, 142). In addition, another *de novo* mutation of *SCN8A* was discovered in a child having epileptic encephalopathy with congenital abnormalities (143).

Prior to this, a loss-of-function frameshift mutation (also identified in parents) caused by a 2 bp deletion p.(Pro1719ArgfsX6) was detected as a possible underlying factor for cerebellar ataxia associated with cognitive problems (144). Another study also identified seven potentially pathogenic variants in *SCN8A*, in patients manifesting seizures and intellectual disorder (142). Four of them were *de novo* and uninherited from any of their parents, another was paternally inherited (father was asymptomatic), while the last two were unknown. Studies on *SCN8A* null mice revealed reduced excitation in the hippocampal neurons (145) while experimental works to overexpress *SCN8A* in excitatory neurons were associated with seizures (146).

The understanding of the role of *SCN8A* in seizure initiation and epilepsy became well-known following the detection of *de novo* mutations in patients having epileptic encephalopathies with varying seizure severities. Another heterozygous mutation c.2300C>T in *SCN8A* was identified in a patient possessing delayed development, cognitive disability, and unmanageable seizures. This produced a mutated Nav1.6 polypeptide with a substituted amino acid p.(T767I) (100). Functional characterization of this mutation further revealed a gain-of-function, with a hyperpolarizing shift (10 mV) in voltage dependence of activation, including a rise in ramp current. These works enhanced the understanding of the gain-of-function roles of *SCN8A* mutations in epileptic encephalopathies, and more importantly, that *SCN8A* is a causative gene in children epilepsies. The works of Pan and Cummins (147) corroborate the gain-of-function theory, thereby suggesting a hyperpolarization-shift as a mechanism of action for the R850Q mutation. They further applied and recommended the use of computational simulation to predict the impact of variants on channel function.

However, loss-of-function mutations had been experimentally implicated in *SCN8A*. de Kovel et al. (148) demonstrated that the loss-of-function missense mutation in Nav1.6 p.(R223G) on inhibitory interneurons is another cause of infantile epileptic encephalopathies. Functional characterization studies showed a loss-of-function depicted by a significant decrease in the amplitude of current. Both gain-of-function mutations affecting Nav1.6 channels on principal excitatory neurons and loss-of-function mutations that affect Nav1.6 channels on inhibitory interneurons may lead to seizure generation.

Sprissler et al. (149) identified a new drug target and expanded the pathogenesis of *SCN8A* encephalopathies by comparing gene expression patterns before and after seizure onset and afterwards in a mouse model of early infantile epileptic encephalopathy Type 13 (149). The mutation, p.(N1768D) was introduced into the genome of the mouse by TALEN targeting. These knock-in mice exactly demonstrated the seizure disorder around the 3rd and 4th months of life (139). In addition, developmental disabilities were expressed in the mice, characterized by skewed postures, impeded movements, and tremors, which are typical of *SCN8A* encephalopathies. This further establish that either gain or loss-of-function mutations in *SCN8A* cause epilepsy. Recent studies show that loss-of-function mutations predispose to cognitive decline and intellectual disability without seizures while gain-of-function mutations result in more severe phenotypes associated with intractable seizures (150). More functional studies (using neurons) that will corroborate the findings of Liu et al. (150) are required to establish these genotype-phenotype relationships.

The pharmacological response of patients with gain-of-function mutations in *SCN8A* has been studied (151). Using a study involving four patients with *SCN8A* mutations, the authors discovered that sodium channel antagonists such as phenytoin at high therapeutic doses effectively manage *SCN8A* seizures. This is true in the case of a Chinese girl bearing the *SCN8A* mutation, p.(N1318S), in whom the administration of sodium channel antagonists proved successful (152).

**Figure 5:**
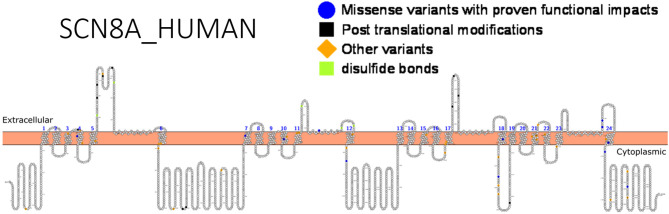
Primary structure of *SCN8A* protein showing regions with functionally validated epilepsy-variants from literature. Regions of missense variants with proven functional impacts (blue), regions of post-translational modifications (PTMs) (black), other variants (orange) and regions of disulfide bonds (lemon). [Created using Protter (78). Visualization link: https://bit.ly/2Vz6AB6].

A difference in drug response can occur in patients with different sodium channel mutations as observed in the cases treatment of *SCN1A* and *SCN8A* related seizures with sodium channel blockers. The former resulted in exacerbated seizures while the latter led to seizure mitigation. Hence, genetic testing is indispensable. Functional studies can help detect gain or loss-of-function variants that can guide drug design, identify high risk, population-related variants that are important for genetic testing and understand relationships between variants and specific drug response. Functional validation has been done for almost 20 different *SCN8A* variants (147). Figure 5 shows *SCN8A* primary structure with regions having variants have been functionally validated from literature while Table 7 shows the affected regions with references.

**Table 7:** Regions of SCN8A with Functionally validated epilepsy variants with references.

**Gene**	**Amino acids affected by missense variants**	**Deletion regions**	**Protein-truncating variant regions**	**References**
***SCN8A***	R850, N984, P1719, N1716, G1451, V929, T767, N1768, R223, R1872, G1475, M1760	-	Pro1719ArgfsX6	(100, 138, 140, 141, 144, 148, 153, 154)

## *SCN9A*: An Epilepsy Gene or Not?

The sodium channel; Nav1.7 is expressed in the brain stem, cerebral cortex and spinal cord, but its major expression is in the peripheral nervous system (PNS) (155). Some *SCN9A* variants have been found in epilepsy and autism spectrum disorders. Also, many mutations in this gene are related to inherited chronic pain syndromes, erythermalgia, familial rectal pain (156, 157). Some *SCN9A* missense mutations have also been reported in patients with DS. Because the first *SCN9A* mutations identified occurred concurrently with *SCN1A* mutations, they were initially categorized as “genes with modifying roles to *SCN1A*” on inhibitory interneurons. But later on, six missense mutations were discovered in patients with febrile seizures not having *SCN1A* mutation, suggesting that *SCN9A* could be a major participant in cryptogenic epilepsies (158). Recently, Zhang et al. (159) discovered a possibly pathogenic missense variant; Y1958C causing GEFS+ in a Chinese family. *In silico* prediction algorithms validated this variant as likely deleterious but functional validation is still required. Due to the sparsity of *SCN9A* mutations in epilepsy, reports classifying it as a true epilepsy gene are inconclusive.

## Genome Wide Association Studies (GWAS) of Sodium Channel Genes Involved in Epilepsy

Some reports from GWAS of epilepsy in different populations has shown some high-risk susceptibility single nucleotide polymorphisms (SNPs) within sodium channel genes (Table 8). These include studies of European, Asian, African-American origin.

**Table 8:** GWAS reports of sodium channel genes involved in epilepsy.

**Gene**	**SNPID**	**Associated phenotype**	**Population studied**	**References**
*SCN1A, SCN1A*-AS1	Rs7587026-A	Mesial temporal lobe epilepsy with hippocampal sclerosis	Europeans	(160)
*SCN1A, SCN1A*-AS1	rs11890028-T	Generalized epilepsy	European	(161)
*SCN1A*	rs6732655-T	All epilepsy	European, Australian, United states, Han Chinese	(162)
*SCN1A*	rs12987787-T	Partial epilepsy	European, Australia, United states, Han Chinese	(162)
*SCN1A*-AS1, *SCN1A*	rs6432860-G	Febrile seizure	European	(163)
*SCN1A*-AS1, *SCN1A*	undefined	Generalized epilepsy	Europeans and African American	(164)
*SCN1A*-AS1, *SCN1A*	rs2212656-A	Partial epilepsy	European, African American, Han Chinese	(164)
*SCN1A*-AS1, *SCN1A*	rs6432877-G	All epilepsy	European, African American, Han Chinese	(164)
*SCN2A*	rs3769955-T	Febrile seizure	European	(163)

In spite of the abundance of studies pointing out the roles of sodium channel genetic mutations in epilepsy, results published on the GWAS catalog (https://www.ebi.ac.uk/gwas/) are still relatively few. Feenstra et al. (163) conducted a genome wide association studies on the genetic causality of febrile seizures in individuals presenting with vaccine-associated and vaccine-unassociated seizures (163). Genetic loci associated with epilepsy (2q24.3) included two different sodium channel genes (rs6432860 in *SCN1A* and rs3769955 in *SCN2A*). More GWA studies in diverse population have confirmed the involvement of the genetic loci, 2q24.3 in both monogenic and polygenic epilepsies. In a cohort study of 8696 cases, the SNP, rs6732655-T was associated with all epilepsy phenotypes while rs12987787-T was associated with focal epilepsies (162). Both SNPs mapped to *SCN1A* within the 2q24.3 genomic loci.

A larger cohort of 15,212 cases identified 16 genomic loci associated with different epilepsy phenotypes (164). Amongst these was the 2q24.3 locus harboring *SCN1A-SCN3A*. The lead SNPs in this region were rs6432877-G for all epilepsies and rs11890028 (G) for genetic generalized epilepsies. The same locus was previously identified as causality for common generalized epilepsies (161). Very large sample sizes are required to increase power and identify more loci in GWAS of epilepsy. As a complex and genetically heterogenous disorder, it is also difficult to measure heritability in polygenic epilepsies. Hence, small cohort sizes (e.g., <3,500 cases) may be unable to establish heritability of rare variants with small effect sizes (165). This warrants the adoption of large cohort sizes. It would be advantageous if many countries would adopt the establishment of biobanks with detailed sampling information, as this would minimize sampling time in large cohorts studies (166).

Many SNPs are expected to have mitigated or aggravated effects when they occur in the presence of other gene mutations (i.e., epistatic interactions). Although GWAS reports may give a clue to the presence of pleiotropic effects amongst genes (164), they do not account for the actual damage-potential of the variants under the influence of mutations from other genes, Hence, necessitating functional studies.

Despite the drawbacks of GWAS, its innate capacity still needs to be harnessed. More research collaborations and funding are required to promote GWAS in regions with high epilepsy burden. GWAS of epilepsy in epilepsy endemic regions are expected to discover ethnically related polymorphisms and genetic diversity of epilepsy amongst different populations (167).

## Conclusion and Future Considerations

Since epilepsy is a polygenic and heterogenous disorder, the increasingly cheap cost of whole genome and exome sequencing is rapidly advancing variant identification in candidate genes, including those acting as disease modifiers. The exponential increase in detected variants has created a huge need for more functional studies to validate the underlying mechanisms. Even though the evolutionary conservation amongst the different α-subunits has prevented the designing of drugs specifically for different VGSC channels, some progress has been recorded in determining effective therapies based on the knowledge of molecular mechanisms of mutations. Meanwhile, the outcome bias introduced in the functional analyses done using heterologous mammalian cells lines should be rapidly eliminated using patient-derived, genetically engineered neurons from hiPSCs or transgenic animals. Moreover, computational methods should be considered to guide decision-making for appropriate variants for functional studies. Above all, the field of epilepsy genetics has experienced a rapid improvement and much understanding has been gained over the past decades.

## Limitations of This Study

Reports reviewed in this article were majorly from European, Asian, and American populations. We observed the absence of genetic studies on epilepsy from African populations, despite the high prevalence of this disorder in Africa. Fodjo et al. (168) reported the prevalence of epilepsy in West Africa as 13.14 per 1,000 persons, Akinsulore and Adewuya (169) reported an incidence rate of 5.3–37 per 100 persons in Nigeria, Esterhuizen et al. (167) reported a median occurrence of 14.2 per 1,000 in Sub-Saharan Africa while Mukuku et al. (170) reported 5.2–74 per 100 persons with epilepsy. Although variances occur depending on the studied region, there is remarkably higher prevalence of epilepsy in low- and middle-income countries (LMICs) than in developed nations (171). Over three-fourths of persons having epilepsy live in the LMICs, and about two-thirds of these cases have unknown etiology (167, 170). Although some reports suggest a relationship between epilepsy and CNS infections in Africa (168, 172), the etiology of most epilepsy remains unknown. This emphasizes the role of genetics and an urgent need for genetic investigations.

## Author Contributions

AI, RS, and AE developed the concept of the manuscript. AI wrote the first draft. RS, AE, AY, and SS reviewed and modified the draft. All authors approved the final version of the work.

## Conflict of Interest

The authors declare that the research was conducted in the absence of any commercial or financial relationships that could be construed as a potential conflict of interest.

## Abbreviations

GABRG2: Gamma-aminobutyric acid receptor, gamma-2
KCNT1: Potassium channel, subfamily T, member 1
KCNQ2: Potassium channel, voltage-gated, KQT-like subfamily, member 2
FOXG1: Forkhead box G1
CDKL5: Cyclin-dependent kinase-like 5
MECP2: Methyl-CpG-binding protein 2
ARX: Aristaless-related homeobox, x-linked
*SCN1A*: Sodium channel, neuronal type I, alpha subunit
*SCN2A*: Sodium channel, voltage-gated, type II, alpha subunit
*SCN3A*: Sodium channel, voltage-gated, type III, alpha subunit
*SCN1B*: Sodium channel, voltage-gated, type I, beta subunit
*SCN8A*: Sodium channel, voltage-gated, type VIII, alpha subunit
*SCN9A*: Sodium channel, voltage-gated, type IX, alpha subunit
STXBP1: Syntaxin-binding protein 1
SLC25A22: Solute carrier family 25 (mitochondrial carrier, glutamate), member 22
PCDH19: Protocadherin 19
SPTAN1: Spectrin, alpha, nonerythrocytic 1
ARHGEF9: Rho guanine nucleotide exchange factor 9.

## References

[1] Boison D. The biochemistry and epigenetics of epilepsy: focus on adenosine and glycine. Front Mol Neurosci. (2016) 9:1–7. 10.3389/fnmol.2016.00026 27147960 PMC4829603

[2] Chen T Giri M Xia Z Subedi YN Li Y. Genetic and epigenetic mechanisms of epilepsy: a review. Neuropsychiatric Dis Treatment. (2017) 13:1841–59. 10.2147/NDT.S142032 28761347 PMC5516882

[3] Falco-walter JJ Scheffer IE Fisher RS. The new definition and classification of seizures and epilepsy. Epilepsy Res. (2018) 139:73–9. 10.1016/j.eplepsyres.2017.11.015 29197668

[4] Scheffer IE Berkovic S Capovilla G Connolly MB French J Guilhoto L. ILAE POSITION PAPER ILAE classification of the epilepsies : position paper of the ILAE Commission for Classification and Terminology. Epilepsia. (2017) 58:512–21. 10.1111/epi.13709 28276062 PMC5386840

[5] Sweet KM Shaw DW Chapman T. Cerebral palsy and seizures in a child with tubulinopathy pattern dysgenesis and focal cortical dysplasia. Radiol Case Rep. (2017) 12:396–400. 10.1016/j.radcr.2016.12.008 28491196 PMC5417618

[6] Escayg A Goldin AL. Sodium channel *SCN1A* and epilepsy: mutations and mechanisms. Epilepsia. (2010) 51:1650–8. 10.1111/j.1528-1167.2010.02640.x 20831750 PMC2937162

[7] Bechi G Rusconi R Cestèle S Striano P Franceschetti S Mantegazza M. Rescuable folding defective NaV1.1 (*SCN1A*) mutants in epilepsy: properties, occurrence, and novel rescuing strategy with peptides targeted to the endoplasmic reticulum. Neurobiol Dis. (2015) 75:100–14. 10.1016/j.nbd.2014.12.028 25576396

[8] Nelson DL Cox MM. Publisher: Susan Winslow, 6th ed. New York, NY: WH Freeman Company (2013).

[9] Claes LR Deprez L Suls A Baets J Smets K Van Dyck T. The *SCN1A* variant database: a novel research and diagnostic tool. Hum Mutat. (2009) 30:E904–20. 10.1002/humu.21083 19585586

[10] Kaplan ID Isom L Petrou S. Role of sodium channels in epilepsy. Cold Spring Harb Perspect Med. (2016) 6:a022814. 10.1101/cshperspect.a022814 27143702 PMC4888813

[11] Kearney JA Plummer NW Smith MR Kapur J Cummins TR Waxman SG. A gain-of-function mutation in the sodium channel gene *SCN2A* results in seizures and behavioral abnormalities. Neurosicnce. (2001) 102:307–17. 10.1016/S0306-4522(00)00479-6 11166117

[12] Holland KD Kearney JA Glauser TA Buck G Keddache M Blankston JR. Mutation of sodium channel *SCN3A* in a patient with cryptogenic pediatric partial epilepsy. Neurosci Lett. (2008) 433:65–70. 10.1016/j.neulet.2007.12.064 18242854 PMC2423278

[13] Catterall WA Kalume F Oakley JC. NaV1.1 channels and epilepsy. J Physiol. (2010) 588:1849–59. 10.1113/jphysiol.2010.187484 20194124 PMC2901973

[14] Brackenbury WJ Isom LL. Na channel beta subunits: overachievers of the ion channel family. Front Pharmacol. (2011) 2:53. 10.3389/fphar.2011.00053 22007171 PMC3181431

[15] Matthews E Labrum R Sweeney MG Sud R Haworth A Chinnery PF. Voltage sensor charge loss accounts for most cases of hypokalemic periodic paralysis. Neurology. (2009) 72:1544–7. 10.1212/01.wnl.0000342387.65477.46 19118277 PMC2848101

[16] Tsujino A Maertens C Ohno K Shen X-M Fukuda T Harper CM. Myasthenic syndrome caused by mutation of the *SCN4A* sodium channel. Proc Nat Acad Sci USA. (2003) 100:7377–82. 10.1073/pnas.1230273100 12766226 PMC165883

[17] Darbar D Kannankeril PJ Donahue BS Kucera G Stubblefield T Haines JL. Cardiac sodium channel (*SCN5A*) variants associated with atrial fibrillation. Circulation. (2008) 117:1927–35. 10.1161/CIRCULATIONAHA.107.757955 18378609 PMC2365761

[18] Makita N Behr E Shimizu W Horie M Sunami A Crotti L. The E1784K mutation in *SCN5A* is associated with mixed clinical phenotype of type 3 long QT syndrome. Clin Invest. (2008) 118:2219–29. 10.1172/JCI34057 18451998 PMC2350431

[19] Albert CM Nam EG Rimm EB Jin HW Hajjar RJ Hunter DJ. Cardiac sodium channel gene variants and sudden cardiac death in women. Circulation. (2008) 117:16–23. 10.1161/CIRCULATIONAHA.107.736330 18071069

[20] Dichgans M Freilinger T Eckstein G Babini E Lorenz-Depiereux B Biskup S. Mutation in the neuronal voltage-gated sodium channel *SCN1A* in familial hemiplegic migraine. Lancet Neurol. (2005) 366:371–6. 10.1016/S0140-6736(05)66786-4 16054936

[21] Schwarz N Bast T Gaily E Golla G Gorman KM Griffiths LR. Clinical and genetic spectrum of *SCN2A*-associated episodic ataxia. Eur J Pediat Neurol. 2019:438–47. 10.1016/j.ejpn.2019.03.001 30928199

[22] Fazeli W Becker K Herkenrath P Duchting C Korber F Landgraf P. Dominant *SCN2A* mutation causes familial episodic ataxia and impairment of speech development. Neuropediatrics. (2018) 49:379–84. 10.1055/s-0038-1668141 30165711

[23] Jurkat-Rott K Mitrovic N Hang C Kouzmekine A Iaizzo P Herzog J. Voltage-sensor sodium channel mutations cause hypokalemic periodic paralysis type 2 by enhanced inactivation and reduced current. Proc Nat Acad Sci USA. (2000) 97:9549–54. 10.1073/pnas.97.17.9549 10944223 PMC16902

[24] Gay S Dupuis D Faivre L Masurel-Paulet A Labenne M Colombani M. Severe neonatal non-dystrophic myotonia secondary to a novel mutation of the voltage-gated sodium channel (*SCN4A*) gene. Am J Med Genet. (2008) 146A:380–3. 10.1002/ajmg.a.32141 18203179

[25] Arnold WD Feldman DH Ramirez S He L Kassar D Quick A. Defective fast inactivation recovery of Na(v)1.4 in congenital myasthenic syndrome. Ann Neurol. (2015) 77:840–50. 10.1002/ana.24389 25707578 PMC4510994

[26] Francis DG Rybalchenko V Struyk A Cannon SC. Leaky sodium channels from voltage sensor mutations in periodic paralysis, but not paramyotonia. Neurology. (2011) 76:1635–41. 10.1212/WNL.0b013e318219fb57 21490317 PMC3100087

[27] Watanabe H Koopmann TT Le Scouarnec S Yang T Ingram CR Schott J-J. Sodium channel beta-1 subunit mutations associated with Brugada syndrome and cardiac conduction disease in humans. J Clin Invest. (2008) 118:2260–8. 10.1172/JCI33891 18464934 PMC2373423

[28] Makita N Sasaki K Groenewegen WA Yokota T Yokoshiki H Murakami T. Congenital atrial standstill associated with coinheritance of a novel *SCN5A* mutation and connexin 40 polymorphisms. Heart Rhythm. (2005) 2:1128–34. 10.1016/j.hrthm.2005.06.032 16188595

[29] Olson TM Michels VV Ballew JD Reyna SP Karst ML Herron KI. Sodium channel mutations and susceptibility of heart failure and atrial fibrillation. JAMA. (2005) 293:447–54. 10.1001/jama.293.4.447 15671429 PMC2039897

[30] Watanabe H Darbar D Kaiser DW Jiramongkolchai K Chopra S Donahue BS. Mutations in sodium channel beta-1- and beta-2-subunits associated with atrial fibrillation. Circ Arrhythm Electrophysiol. (2009) 2:268–78. 10.1161/CIRCEP.108.779181 19808477 PMC2727725

[31] Wagnon JL Barker BS Ottolini M Park Y Volkheimer A Valdez P. Loss-of-function variants of *SCN8A* in intellectual disability without seizures. Neurol Genet. (2017) 3:1–6. 10.1212/NXG.0000000000000170 28702509 PMC5499976

[32] Michiels JJ te Morsche RHM Jansen JBMJ Drenth JPH. Autosomal dominant erythermalgia associated with a novel mutation in the voltage-gated sodium channel alpha-subunit Na(v)1.7. Arch Neurol. (2005) 62:1587–90. 10.1001/archneur.62.10.1587 16216943

[33] Cox JJ Reimann F Nicholas AK Thornton G Roberts E Springell K. An *SCN9A* channelopathy causes congenital inability to experience pain. Nature. (2006) 444:894–8. 10.1038/nature05413 17167479 PMC7212082

[34] Yuan J Matsuura E Higuchi Y Hashiguchi A Nakamura T Nozuma S. Hereditary sensory and autonomic neuropathy type IID caused by an *SCN9A* mutation. Neurology. (2013) 80:1641–9. 10.1212/WNL.0b013e3182904fdd 23596073

[35] Meglic A Perkovic-Benedik M Trebusak Podkrajsek K Bertok S. Painful micturition in a small child: an unusual clinical picture of paroxysmal extreme pain disorder. Pediat Nephrol. (2014) 29:1643–6. 10.1007/s00467-014-2819-2 24817410

[36] Devigili G Eleopra R Pierro T Lombardi R Rinaldo S Lettieri C. Paroxysmal itch caused by gain-of-function Na(v)1.7 mutation. Pain. (2014) 155:1702–7. 10.1016/j.pain.2014.05.006 24820863

[37] Lombardi R Dong J Rodriguez G Al. E. Genetic fate mapping identifies second heart field progenitor cells as a source of adipocytes in arrhythmogenic right ventricular cardiomyopathy. Circ Res. (2009) 104:1076–84. 10.1161/CIRCRESAHA.109.196899 19359597 PMC2767296

[38] Meadows L Isom L. Sodium channels as macromolecular complexes: implications for inherited arrhythmia syndromes. Cardiovasc Res. (2005) 67:448–58. 10.1016/j.cardiores.2005.04.003 15919069

[39] Chen S Gurha P Lombardi R. The hippo pathway is activated and is a causal mechanism for adipogenesis in arrhythmogenic cardiomyopathy. Circ Res. (2014) 114:454–68. 10.1161/CIRCRESAHA.114.302810 24276085 PMC3946717

[40] Dutton SB Makinson CD Papale LA Shankar A Balakrishnan B Nakazawa K. Preferential inactivation of *SCN1A* in parvalbumin interneurons increases seizure susceptibility. Neurobiol Dis. (2012) 49C:211–20. 10.1016/j.nbd.2012.08.012 22926190 PMC3740063

[41] Du J Simmons S Brunklaus A Adiconis X Hession CC Fu Z. Differential excitatory vs inhibitory SCN expression at single cell level regulates brain sodium channel function in neurodevelopmental disorders. Eur J Paediatric Neurol. (2020) 24:129–33. 10.1016/j.ejpn.2019.12.019 31928904

[42] Oliva MK McGarr TC Beyer BJ Gazina E Kaplan DI Cordeiro L. Physiological and genetic analysis of multiple sodium channel variants in a model of genetic absence epilepsy. Neurobiol Dis. (2014) 67:180–90. 10.1016/j.nbd.2014.03.007 24657915 PMC4298829

[43] Lorincz A Nusser Z. Molecular identity of dendritic voltage gated sodium channels. Science. (2010) 328:906–9. 10.1126/science.1187958 20466935 PMC3546315

[44] Xie Y Ng NN Safrina OS Ramos CM Ess KC Schwartz PH. Comparisons of dual isogenic human iPSC pairs identify functional alterations directly caused by an epilepsy associated *SCN1A* mutation. Neurobiol Dis. (2020) 134:104627. 10.1016/j.nbd.2019.104627 31786370

[45] Surovy M Soltysova A Kolnikova M Sykora P Ilencikova D Ficek A. Novel *SCN1A* variants in Dravet syndrome and evaluating a wide ap- proach of patient selection. Gen Physiol Biophys. (2016) 35:333–42. 10.4149/gpb_2016002 27045673

[46] Hirose S Scheffer IE Marini C De Jonghe PAE Goldman AM Kauffman M. *SCN1A* testing for epilepsy: application in clinical practice. Epilepsia. (2013) 54:946–52. 10.1111/epi.12168 23586701

[47] Marini C Scheffer IE Nabbout R Suls A De Jonghe P Zara F. The genetics of Dravet syndrome. Epilepsia. (2011) 52:24–9. 10.1111/j.1528-1167.2011.02997.x 21463275

[48] Liu Y Lopez-Santiago LF Yuan Y Jones JM Zhang H O'Malley HA. Dravet syndrome patient-derived neurons suggest a novel epilepsy mechanism. Ann Neurol. (2013) 74:128–39. 10.1002/ana.23897 23821540 PMC3775921

[49] Meisler MH O'Brien JE. Gene interactions and modifiers in epilepsy. In: (US) NCfBI editor. In: Noebels JL Avoli M Rogawski MA Olsen RW Delgado-Escueta AV editors. Jasper's Basic Mechanisms of the Epilepsies. 4th ed. Oxford: Oxford University Press (2012). p. 1–12. 10.1093/med/9780199746545.003.0059

[50] Lossin C Wang DW Rhodes TH Vanoye CG George ALJ. Molecular basis of an inherited epilepsy. Neuron. (2002) 34:877–84. 10.1016/S0896-6273(02)00714-6 12086636

[51] Lossin C Rhodes TH Desai RR Vanoye CG Wang D Carniciu S. Epilepsy-associated dysfunction in the voltagegated neuronal sodium channel *SCN1A*. J Neurosci. (2003) 23:11289–95. 10.1523/JNEUROSCI.23-36-11289.2003 14672992 PMC6740520

[52] Tang B Dutt K Papale L Rusconi R Shankar A Hunter J. A BAC transgenic mouse model reveals neuron subtype-specific effects of a Generalized Epilepsy with Febrile Seizures Plus (GEFS+) mutation. Neurobiol Dis. (2009) 35:91–102. 10.1016/j.nbd.2009.04.007 19409490 PMC2735447

[53] Kluckova D Kolnikova M Lacinova L Jurkovicova-Tarabova B Foltan T Demko V. A Study among the genotype, functional alternations, and phenotype of 9 *SCN1A* mutations in epilepsy patients. Sci Rep. (2020) 10:1–13. 10.1038/s41598-020-67215-y 32581296 PMC7314844

[54] Lossin C. A catalog of *SCN1A* variants. Brain Dev. (2009) 31:114–30. 10.1016/j.braindev.2008.07.011 18804930

[55] Zhang YH Sun HH Liu XY Ma XW Yang ZX Xiong H. Clinical features and *SCN1A* gene mutation analysis of severe myoclonic epilepsy of infancy. Zhonghua er ke za zhi. (2008) 46:769–73. 19099883

[56] Zucca C Redaelli F Epifanio R Zanotta N Romeo A Lodi M. Cryptogenic epileptic syndromes related to *SCN1A*. Twelve novel mutations identified. Arch Neurol. (2008) 65:489–94. 10.1001/archneur.65.4.489 18413471

[57] Heron SE Scheffer IE Grinton BE Eyre H Oliver KL Bain S. Familial neonatal seizures with intellectual disability caused by a microduplication of chromosome 2q24. Epilepsia. (2010) 51:1865–9. 10.1111/j.1528-1167.2010.02558.x 20384724

[58] Chen YJ Shi YW Xu HQ Chen ML Gao MM Sun WW. Electrophysiological differences between the same pore region mutation in *SCN1A* and *SCN3A*. Mol Neurobiol. (2015) 51:1263–70. 10.1007/s12035-014-8802-x 24990319

[59] Tunçer GÖ Teber S Albayrak P Kutluk MG Deda G. A case of Dravet Syndrome with a newly defined mutation in the *SCN1A* gene. Turkish Archiv Pediatr. (2018) 53:259. 10.5152/TurkPediatriArs.2018.4197 30872930 PMC6408181

[60] Shi L Zhu M Li H Wen Z Chen X Luo J. *SCN1A* and *SCN2A* polymorphisms are associated with response to valproic acid in Chinese epilepsy patients. Eur J Clin Pharmacol. (2019) 2019:1–9. 10.1007/s00228-019-02633-0 30693367

[61] Ottman R Hirose S Jain S Lerche H Lopes-Cendes I Noebels Jea JL. Genetic testing in the epilepsies - report of the ILAE Genetics Commission. Epilepsia. (2010) 51:655–70. 10.1111/j.1528-1167.2009.02429.x 20100225 PMC2855784

[62] Fang ZX Hong SQ Li TS Wang J Xie LL Han W. Genetic and phenotypic characteristics of *SCN1A*-related epilepsy in Chinese children. NeuroReport. (2019) 2019:1259. 10.1097/WNR.0000000000001259 31009440

[63] Krikova E Val'dman E Avakian GN AndreevIaA EV Denisov FK Rider RR. Association study of the SCN1 gene polymorphism and effective dose of lamotrigine. Zhurnal Nevrologii i Psikhiatrii Imeni SS Korsakova. (2009) 109:57–62. 20037572

[64] Cross JH Devinsky O Marsh E Miller I Nabbout R Scheffer IE. Cannabidiol (CBD) reduces convulsive seizure frequency in Dravet syndrome: results of a multi-center, randomized, controlled trial. Epilepsia. (2017) 58:S12. 10.1111/epi.13944

[65] Eschbach K Knupp KG. Stiripentol for the treatment of seizures in Dravet syndrome. Expert Rev Clin Pharmacol. (2019) 12:379–88. 10.1080/17512433.2019.1605904 31017478

[66] Ceulemans B Boel M Leyssens K Van Rossem C Neels P Jorens PG. Successful use of fenfluramine as an add-on treatment for Dravet syndrome. Epilepsia. (2012) 53:1131–9. 10.1111/j.1528-1167.2012.03495.x 22554283

[67] Ceulemans B Schoonjans AS Marchau F Paelinck BP Lagae L. Five-year extended follow-up status of 10 patients with Dravet syndrome treated with fenfluramine. Epilepsia. (2016) 57:e129–34. 10.1111/epi.13407 27197941

[68] Schoonjans A Paelinck BP Marchau F Gunning B Gammaitoni A Galer BS. Low-dose fenfluramine significantly reduces seizure frequency in Dravet syndrome: a prospective study of a new cohort of patients. Eur J Neurol. (2017) 24:309–14. 10.1111/ene.13195 27790834 PMC5298030

[69] Schoonjans AS Marchau F Paelinck BP Lagae L Gammaitoni A Pringsheim M. Cardiovascular safety of low-dose fenfluramine in Dravet syndrome: a review of its benefit-risk profile in a new patient population. Curr Med Res Opin. (2017) 33:1773–81. 10.1080/03007995.2017.1355781 28704161

[70] Lagae L Sullivan J Knupp K Laux L Polster T Nikanorova M. Fenfluramine hydrochloride for the treatment of seizures in Dravet syndrome: a randomised, double-blind, placebo-controlled trial. Lancet. (2019) 394:2243–54. 10.1016/S0140-6736(19)32500-0 31862249

[71] Guo F Yu N Cai J-q Quinn T Zong Z-h Zeng Y-j. Voltage-gated sodium channel NAv1.1, Nav1.3 and β1 subunit were up-regulated in the hippocampus of spontaneously epileptic rat. Brain Res Bulletin. (2008) 75:179–87. 10.1016/j.brainresbull.2007.10.005 18158113

[72] Younus I Reddy DS. Epigenetic interventions for epileptogenesis: a new frontier for curing epilepsy. Pharmacol Therapeut. (2017) 177:108–22. 10.1016/j.pharmthera.2017.03.002 28279785 PMC5565684

[73] Jiao J Yuanyuan Y Yiwu S Jiayu C Ru G Yong F. Modeling Dravet syndrome using induced pluripotent stem cells (iPSCs) and directly converted neurons. Hum Mol Genet. (2013) 22:4241–52. 10.1093/hmg/ddt275 23773995

[74] Higurashi N Uchida T Lossin C Misumi Y Okada Y Akamatsu W. A human Dravet syndrome model from patient induced pluripotent stem cells. Mol Brain. (2013) 6:19. 10.1186/1756-6606-6-19 23639079 PMC3655893

[75] Sun Y Paşca SP Portmann T Goold C Worringer KA Guan W. A deleterious Nav1.1 mutation selectively impairs telencephalic inhibitory neurons derived from Dravet syndrome patients. Elife. (2016) 5:e13073. 10.7554/eLife.13073 27458797 PMC4961470

[76] Liu J Gao C Chen W Ma W Li X Shi Y. CRISPR/Cas9 facilitates investigation of neural circuit disease using human iPSCs: mechanism of epilepsy caused by an *SCN1A* loss-of-function mutation. Transl Psychiatry. (2016) 6:e703. 10.1038/tp.2015.203 26731440 PMC5068877

[77] Zhao GX Zhang Z Cai WK Shen ML Wang P He GH. Associations between CYP3A4, CYP3A5 and *SCN1A* polymorphisms and carbamazepine metabolism in epilepsy: a meta-analysis. medRxiv. (2020). 10.1101/2020.03.03.20030783 33756436

[78] Omasits U Ahrens CH Müller S Wollscheid B. Protter: interactive protein feature visualization and integration with experimental proteomic data. Bioinformatics. (2013) 30:884–6. 10.1093/bioinformatics/btt607 24162465

[79] Wallace RH Wang DW Singh R Scheffer IE George ALJ Phillips HA. Febrile seizures and generalized epilepsy associated with a mutation in the Naþ-channel beta1 subunit gene *SCN1B*. Nat Genetics. (1998) 19:366e70. 10.1038/1252 9697698

[80] Baroni D Barbieri R Picco C Moran O. Functional modulation of voltage-dependent sodium channel expression by wild type and mutated C121W-β1 subunit. J Bioenerg Biomembr. (2013) 45:45:353–68. 10.1007/s10863-013-9510-3 23584539

[81] O'Malley HA Isom LL. Sodium channel β subunits : emerging targets in channelopathies. Annu Rev Physiol. (2015) 77:481–504. 10.1146/annurev-physiol-021014-071846 25668026 PMC4817109

[82] Shimizu H Miyazaki H Ohsawa N Shoji S Ishizuka-Katsura Y Tosaki A. Structure-based site-directed photo-crosslinking analyses of multimeric cell-adhesive interactions of voltage-gated sodium channel β subunits. Sci Rep. (2016) 6:26618. 10.1038/srep26618 27216889 PMC4877568

[83] Patino GA Claes LRF Lopez-Santiago LF Slat EA Dondeti RSR Chen C. A functional null mutation of *SCN1B* in a patient with Dravet syndrome. J Neurosci. (2009) 29:10764–78. 10.1523/JNEUROSCI.2475-09.2009 19710327 PMC2749953

[84] Scheffer IE Harkin LA Grinton BE Dibbens LM Turner SJ Zielinski MA. Temporal lobe epilepsy and GEFS+ phenotypes associated with *SCN1B* mutations. Brain. (2007) 130:100–9. 10.1093/brain/awl272 17020904

[85] Ogiwara I Nakayama T Yamagata T Ohtani H Mazaki E Tsuchiya S. A homozygous mutation of voltage-gated sodium channel beta(I) gene *SCN1B* in a patient with Dravet syndrome. Epilepsy. (2012) 53:e200e3. 10.1111/epi.12040 23148524

[86] Aman TK Grieco-Calub TM Chen C Rusconi R Slat EA Isom LL. Regulation of persistent Na current by interactions between beta subunits of voltage-gated Na channels. J Neurosci. (2009) 29:2027e42. 10.1523/JNEUROSCI.4531-08.2009 19228957 PMC2667244

[87] Kruger LC O'Malley HA Hull JM Kleeman A Patino GA Isom LL. β1-C121W is down but not out: epilepsy-associated *SCN1B*-C121W results in a deleterious gain-of-function. J Neurosci. (2016) 36:6213–24. 10.1523/JNEUROSCI.0405-16.2016 27277800 PMC4899524

[88] Lucas PT Meadows LS Nicholls J Ragsdale DS. An epilepsy mutation in the beta1 subunit of the voltage-gated sodium channel results in reduced channel sensitivity to phenytoin. Epilepsy Res. (2005) 64:77–84. 10.1016/j.eplepsyres.2005.03.003 15922564

[89] Patino GA Brackenbury WJ Bao Y Lopez-santiago LF Malley HAO Chen C. Voltage-gated Na channel β1B : a secreted cell adhesion molecule involved in human epilepsy. J Neurosci. (2011) 31:14577–91. 10.1523/JNEUROSCI.0361-11.2011 21994374 PMC3212034

[90] Marionneau C Carrasquillo Y Norris AJ Townsend RR Isom LL Link AJ. The sodium channel accessory subunit Navb1 regulates neuronal excitability through modulation of repolarizing voltage-gated Kþ channels. J Neurosci. (2012) 32:5716e27. 10.1523/JNEUROSCI.6450-11.2012 22539834 PMC3347704

[91] Nguyen HM Miyazaki H Hoshi N Smith BJ Nukina N Goldin AL. Modulation of voltage-gated K+ channels by the sodium channel beta1 subunit. Proc Natl Acad Sci USA. (2012) 109:18577–82. 10.1073/pnas.1209142109 23090990 PMC3494885

[92] Aeby A Sculier C Bouza AA Askar B Lederer D Schoonjans AS. *SCN1B*-linked early infantile developmental and epileptic encephalopathy. Ann Clin Transl Neurol. (2019) 6:2354–67. 10.1002/acn3.50921 31709768 PMC6917350

[93] Dang LT Quinonez SC Becka BR Isom LL Joshi SM. Dramatic improvement in seizures with phenytoin treatment in an individual with refractory epilepsy and a *SCN1B* variant. Pediatric Neurol. (2020) 108:121–2. 10.1016/j.pediatrneurol.2020.03.012 32303391 PMC8649903

[94] Audenaert D Claes L Ceulemans B Lofgren A Van Broeckhoven C De Jonghe P. A deletion in *SCN1B* is associated with febrile seizures and early-onset absence epilepsy. Neurology. (2003) 61:854–6. 10.1212/01.WNL.0000080362.55784.1C 14504340

[95] Baroni D Picco C Moran O. A mutation of *SCN1B* associated with GEFS+ causes functional and maturation defects of the voltage-dependent sodium channel. Hum Mutat. (2018) 39:1402-15. 10.1002/humu.23589 29992740

[96] Ramadan W Patel N Anazi S Kentab AY Bashiri FA Hamad MH. Confirming the recessive inheritance of *SCN1B* mutations in developmental epileptic encephalopathy. Clin Genet. (2017) 92:327–31. 10.1111/cge.12999 28218389

[97] Tammaro P Conti F Moran O. Modulation of sodium current in mammalian cells by an epilepsy-correlated beta-1-subunit mutation. Biochem Biophys Res Commun. (2002) 291:1095–101. 10.1006/bbrc.2002.6570 11866477

[98] Reid CA Berkovic SF Petrou S. Mechanisms of human inherited epilepsies. Progr Neurobiol. (2009) 87:41–57. 10.1016/j.pneurobio.2008.09.016 18952142

[99] Liao Y Anttonen A Liukkonen E Gaily E Maljevic S Schubert S. *SCN2A* mutation associated with neonatal epilepsy, late-onset episodic ataxia, myoclonus, and pain. Neurology. (2010) 75:1454–8. 10.1212/WNL.0b013e3181f8812e 20956790

[100] Estacion M O'Brien JE Conravey A Hammer MF Waxman SG Dib-Hajj SD. A novel *de novo* mutation of *SCN8A* (Nav1.6) with enhanced channel activation in a child with epileptic encephalopathy. Neurobiol Dis. (2014) 69:117–23. 10.1016/j.nbd.2014.05.017 24874546 PMC4124819

[101] Ogiwara I Ito K Sawaishi Y Osaka H Mazaki E Inoue I. *De novo* mutations of voltage-gated sodium channel alphaII gene *SCN2A* in intractable epilepsies. Neurology. (2009) 73:1046–53. 10.1212/WNL.0b013e3181b9cebc 19786696 PMC2754324

[102] Shi X Yasumoto S Nakagawa E Fukasawa T Uchiya S Hirose S. Missense mutation of the sodium channel gene *SCN2A* causes Dravet syndrome. Brain Dev. (2009) 31:758–62. 10.1016/j.braindev.2009.08.009 19783390

[103] Baasch AL Hüning I Gilissen C Klepper J Veltman JA Gillessen-Kaesbach G. Exome sequencing identifies a *de novo SCN2A* mutation in a patient with intractable seizures, severe intellectual disability, optic atrophy, muscular hypotonia, and brain abnormalities. Epilepsia. (2014) 55:25–9. 10.1111/epi.12554 24579881

[104] Rauch A Wieczorek D Graf E Wieland T Endele S Schwarzmayr T. Range of genetic mutations associated with severe non-syndromic sporadic intellectual disability: an exome sequencing study. Lancet. (2012) 380:1674–82. 10.1016/S0140-6736(12)61480-9 23020937

[105] Kamiya K Kaneda M Sugawara T Mazaki E Okamura N Montal M. A nonsense mutation of the sodium channel gene *SCN2A* in a patient with intractable epilepsy and mental decline. J Neurosci. (2004) 24:2690–8. 10.1523/JNEUROSCI.3089-03.2004 15028761 PMC6729532

[106] Shi X Yasumoto S Kurahashi H Nakagawa E Fukasawa T Uchiya S. Clinical spectrum of *SCN2A* mutations. Brain Dev. (2012) 34:541–5. 10.1016/j.braindev.2011.09.016 22029951

[107] Nakamura K Kato M Osaka H Yamashita S Nakagawa E Haginoya K. Clinical spectrum of *SCN2A* mutations expanding to Ohtahara syndrome. Neurology. (2013) 81:1–8. 10.1212/WNL.0b013e3182a43e57 23935176

[108] Saitoh M Ishii A Ihara Y Hoshino A Terashima H Kubotac M. Missense mutations in sodium channel *SCN1A* and *SCN2A* predispose children to encephalopathy with severe febrile seizures. Epilepsy Res. (2015) 117:1–6. 10.1016/j.eplepsyres.2015.08.001 26311622

[109] Liao Y Deprez L Maljevic S Pitsch J Claes L Hristova D. Molecular correlates of age-dependent seizures in an inherited neonatal-infantile epilepsy. Brain. (2010) 133:1403–14. 10.1093/brain/awq057 20371507

[110] Xu R Thomas EA Jenkins M Gazina EV Chiu C Heron SE. A childhood epilepsy mutation reveals a role for developmentally regulated splicing of a sodium channel. Mol Cell Neurosci. (2007) 35:292–301. 10.1016/j.mcn.2007.03.003 17467289

[111] Barcia G Fleming M Deligniere A Gazula V-R Brown MR Langouet M. *De novo* gainof- function KCNT1 channel mutations cause malignant migrating partial seizures of infancy. Nat Genet. (2012) 44:1255–9. 10.1038/ng.2441 23086397 PMC3687547

[112] Howell KB Mcmahon JM Mackay MT Rodriguez-V Clark D Freeman JL. *SCN2A* encephalopathy A major cause of epilepsy of infancy with migrating focal seizures. Neurology. (2015) 85:1–10. 10.1212/WNL.0000000000001926 26291284 PMC4567464

[113] Matalon D Goldberg E Medne L Marsh ED. Confirming an expanded spectrum of *SCN2A* mutations : a case series. Epileptic Disord. (2014) 16:13–8. 10.1684/epd.2014.0641 24659627

[114] Zeng Q Zhang Y Yang X Zhang J Liu A Liu X. Phenotype study of *SCN2A* gene related epilepsy. Zhonghua Er Ke Za Zhi. (2018) 56:518–23. 10.3760/cma.j.issn.0578-1310.2018.07.009 29996185

[115] Foster LA Johnson MR Macdonald JT Karachunski PI Henry TR Nascene DR. Infantile epileptic encephalopathy associated with *SCN2A* mutation responsive to oral mexiletine. Pediatric Neurol. (2016) 16:1–11. 10.1016/j.pediatrneurol.2016.10.008 27867041

[116] Wolff M Johannesen K Hedrich U Masnada S Rubboli G Gardella Eea. Genetic and phenotypic heterogeneity suggest therapeutic implications in *SCN2A*-related disorders. Brain. (2017) 2017:awx054. 10.1093/brain/awx054 28379373

[117] Wong VCN Fung CW Kwong AKY. *SCN2A* mutation in a Chinese boy with infantile spasm - response to Modified Atkins Diet. Brain Dev. (2015) 37:729–32. 10.1016/j.braindev.2014.10.008 25459969

[118] Su DJ Lu JF Lin LJ Liang JS Hung KL. *SCN2A* mutation in an infant presenting with migrating focal seizures and infantile spasm responsive to a ketogenic diet. Brain Dev. (2018) 40:8724–7. 10.1016/j.braindev.2018.03.005 29625812

[119] Turkdogan D Thomas G Demirel B. Ketogenic diet as a successful early treatment modality for *SCN2A* mutation. Brain Dev. (2019) 41:389–91. 10.1016/j.braindev.2018.10.015 30415926

[120] Lu Y Su Q Li M Dayimu A Dai X Wang Z. Association of *SCN1A, SCN2A*, and UGT2B7 polymorphisms with responsiveness to valproic acid in the treatment of epilepsy. BioMed Res Int. (2020) 2020:1–8. 10.1155/2020/8096235 32185219 PMC7063186

[121] Misra SN Kahlig KM George ALJ. Impaired NaV1.2 function and reduced cell surface expression in benign familial neonatal-infantile seizures. Epilepsia. (2008) 49:1535–45. 10.1111/j.1528-1167.2008.01619.x 18479388 PMC3647030

[122] Kile KB Tian N Durand DM. *SCN2A* sodium channel mutation results in hyperexcitability in the hippocampus *in vitro*. Epilepsia. (2008) 49:488–99. 10.1111/j.1528-1167.2007.01413.x 18031550 PMC2720056

[123] Vanoye CG Gurnett CA Holland KD George AL Kearney JA. Novel *SCN3A* variants associated with focal epilepsy in children. Neurobiol Dis. (2014) 62:313–22. 10.1016/j.nbd.2013.10.015 24157691 PMC3877720

[124] Estacion M Gasser A Dib-Hajj SD Waxman SG. A sodium channel mutation linked to epilepsy increases ramp and persistent current of Nav1.3 and induces hyperexcitability in hippocampal neurons. Exp Neurol. (2010) 224:362–8. 10.1016/j.expneurol.2010.04.012 20420834

[125] Lamar T Vanoye CG Calhoun J Wong JC Dutton SBB Jorge BS. *SCN3A* deficiency associated with increased seizure susceptibility. Neurobiol Dis. (2017) 102:38–48. 10.1016/j.nbd.2017.02.006 28235671 PMC5446790

[126] Zaman T Helbig I BoŽović IB DeBrosse SD Bergqvist AC Wallis K. Mutations in *SCN3A* cause early infantile epileptic encephalopathy. Ann Neurol. (2018) 83:703–17. 10.1002/ana.25188 29466837 PMC5912987

[127] Yoshitomi S Takahashi Y Ishizuka M Yamaguchi T. Three patients manifesting early infantile epileptic spasms associated with 2q24 microduplications. Brain Dev. (2015) 37:874–9. 10.1016/j.braindev.2015.03.001 25843248

[128] Thuresson A-C Van Buggenhout G Sheth F Kamate M Andrieux J Clayton SJ. Whole gene duplication of *SCN2A* and *SCN3A* is associated with neonatal seizures and a normal intellectual development. Clin Genet. (2016) 91:106–10. 10.1111/cge.12797 27153334

[129] Chong FP Saitsu H Sakai Y Imagi T Nakamura R Matsukura M. Deletions of *SCN2A* and *SCN3A* genes in a patient with West syndrome and autistic spectrum disorder. Seizure Eur J Epilepsy. (2018) 60:91–3. 10.1016/j.seizure.2018.06.012 29929112

[130] Wang Y Du X Bin R Yu S Xia Z Zheng G. Genetic variants identified from epilepsy of unknown etiology in Chinese children by targeted exome sequencing. Sci Rep. (2017) 7:1–10. 10.1038/srep46520 28074849 PMC5225856

[131] Larsen J Carvill GL Gardella E Kluger G Schmiedel G Barisic N. The phenotypic spectrum of *SCN8A* encephalopathy. Neurology. (2015) 84:480–9. 10.1212/WNL.0000000000001211 25568300 PMC4336074

[132] Hammer MF Wagnon JL Mefford HC Meisler MH. *SCN8A*-related epilepsy with encephalopathy. In: Pagon RA Adam MP Ardinger HH Wallace SE Amemiya A Bean LJH. editors. Gene Reviews. Seattle: University of Washington (2016) 1993–2021.

[133] Sills GJ. Classical mechanisms of action of antiepileptic drugs. In: Potschka H Lerche H editors, Therapeutic Targets and Perspectives in the Pharmacological Treatment of Epilepsy. Bremen: UNI-MED Verlag. (2013). p. 62–5.

[134] Vacher H Mohapatra DP Trimmer JS. Localization and targeting of voltagedependent ion channels in mammalian central neurons. Physiol Rev. (2008) 88:1407–47. 10.1152/physrev.00002.2008 18923186 PMC2587220

[135] O'Brien JE Meisler MH. Sodium channel *SCN8A* (Nav1.6): properties and *de novo* mutations in epileptic encephalopathy and intellectual disability. Front Genet. (2013) 4:213. 10.3389/fgene.2013.00213 24194747 PMC3809569

[136] Sharkey LM Cheng X Drews V Buchner DA Jones JM Justice MJ. The ataxia3 mutation in the N-terminal cytoplasmic domain of sodium channel Nav1.6 disrupts intracellular trafficking. J Neurosci. (2009) 29:2733–41. 10.1523/JNEUROSCI.6026-08.2009 19261867 PMC2679640

[137] Kearney JA Buchner DA de Haan G Adamska M Levin SI Furay AR. Molecular and pathological effects of a modifier gene on deficiency of the sodium channel *SCN8A* (Nav1.6). Hum Mol Genet. (2002) 11:2765–75. 10.1093/hmg/11.22.2765 12374766

[138] Papale LA Beyer B Jones JM Sharkey LM Tufik S Epstein M. Heterozygous mutations of the voltage-gated sodium channel *SCN8A* are associated with spikewave discharges and absence epilepsy in mice. Hum Mol Genet. (2009) 18:1633–41. 10.1093/hmg/ddp081 19254928 PMC2667290

[139] Wagnon JL Barker BS Hounshell JA Haaxma CA Shealy A Moss T. Pathogenic mechanism of recurrent mutations of *SCN8A* in epileptic encephalopathy. Ann Clin Transl Neurol. (2016) 3:114–23. 10.1002/acn3.276 26900580 PMC4748308

[140] Blanchard MG Willemsen MH Walker JB Dib-Hajj SD Waxman SG Jongmans. *De novo* gain-of-function and lossof- function mutations of *SCN8A* in patients with intellectual disabilities and epilepsy. J Med Genet. (2015) 52:330–7. 10.1136/jmedgenet-2014-102813 25725044 PMC4413743

[141] Veeramah KR O'Brien JE Meisler MH Cheng X Dib-Hajj SD Waxman SG. *de novo* pathogenic *SCN8A* mutation identified by whole-genome sequencing of a family quartet affected by infantile epileptic encephalopathy and SUDEP. Am J Hum Genet. (2012) 90:502–10. 10.1016/j.ajhg.2012.01.006 22365152 PMC3309181

[142] Carvill GL Heavin SB Yendle SC McMahon JM O'Roak BJ Cook J. Targeted resequencing in epileptic encephalopathies identifies *de novo* mutations in CHD2 and SYNGAP1. Nat Genet. (2013) 45:825. 10.1038/ng.2646 23708187 PMC3704157

[143] Vaher U Nõukas M Nikopensius T Kals M Annilo T Nelis M. *De novo SCN8A* mutation identified by whole-exome sequencing in a boy with neonatal epileptic encephalopathy, multiple congenital anomalies, and movement disorders. J Child Neurol. (2013) 29:NP202–6. 10.1177/0883073813511300 24352161

[144] Trudeau MM Dalton JC Day JW Ranum LP Meisler MH. Heterozygosity for a protein truncation mutation of sodium channel *SCN8A* in a patient with cerebellar atrophy, ataxia, and mental retardation. J Med Genet. (2006) 43:527–30. 10.1136/jmg.2005.035667 16236810 PMC2564538

[145] Royeck M Horstmann MT Remy S Reitze M Yaari Y Beck H. Role of axonal NaV1.6 sodium channels in action potential initiation of CA1 pyramidal neurons. J Neurophysiol. (2008) 100:2361–80. 10.1152/jn.90332.2008 18650312

[146] Sun W Wagnon JL Mahaffey CL Briese M Ule J Frankel WN. Aberrant sodium channel activity in the complex seizure disorder of Celf4 mutant mice. J Physiol. (2013) 591:241–55. 10.1113/jphysiol.2012.240168 23090952 PMC3630783

[147] Pan Y Cummins TR. Distinct functional alterations in *SCN8A* epilepsy mutant channels. J Physiol. (2020) 598:381–401. 10.1113/JP278952 31715021 PMC7216308

[148] de Kovel CGF Meisler MH Brilstra EH van Berkestijn FMC Slot Rvt van Lieshout S. Characterization of a *de novo SCN8A* mutation in a patient with epileptic encephalopathy. Epilepsy Res. (2014) 108:1511–8. 10.1016/j.eplepsyres.2014.08.020 25239001 PMC4490185

[149] Sprissler RS Wagnon JL Bunton-Stasyshyn RK Meisler MH Hammer MF. Altered gene expression profile in a mouse model of *SCN8A* encephalopathy. Exp Neurol. (2017) 288:134–41. 10.1016/j.expneurol.2016.11.002 27836728 PMC5215827

[150] Liu Y Schubert J Sonnenberg L Helbig KL Hoei-Hansen CE Koko M. Neuronal mechanisms of mutations in *SCN8A* causing epilepsy or intellectual disability. Brain. (2019) 142:376–90. 10.1093/brain/awy326 30615093

[151] Boerma RS Braun KP van de Broek MP van Berkestijn FM Swinkels ME Hagebeuk EO. Remarkable phenytoin sensitivity in 4 children with *SCN8A*-related epilepsy: a molecular neuropharmacological approach. Neurotherapeutics. (2016) 13:192–7. 10.1007/s13311-015-0372-8 26252990 PMC4720675

[152] Lin KM Su G Wang F Zhang X Wang Y Ren J. A *de novo SCN8A* heterozygous mutation in a child with epileptic encephalopathy: a case report. BMC Pediatrics. (2019) 19:1–6. 10.1186/s12887-019-1796-9 31672125 PMC6824109

[153] Meisler MH. *SCN8A* encephalopathy: mechanisms and models. Epilepsia. (2019) 60:S86–91. 10.1111/epi.14703 31904118 PMC6953611

[154] Martin MS Tang B Papale LA Yu FH Catterall WA Escayg A. The voltage-gated sodium channel *SCN8A* is a genetic modifier of severe myoclonic epilepsy of infancy. Hum Mol Genet. (2007) 16:2892–9. 10.1093/hmg/ddm248 17881658

[155] Toledo-Aral J Moss B He Z Koszowski A Whisenand T Levinson S. Identification of PN1, a predominant voltage-dependent sodium channel expressed principally in peripheral neurons. Proc Natl Acad Sci USA. (1997) 94:1527–32. 10.1073/pnas.94.4.1527 9037087 PMC19825

[156] Cummins TR Dib-Hajj SD Waxman SG. Electrophysiological properties of mutant Nav1.7 sodium channels in a painful inherited neuropathy. J Neurosci. (2004) 24:8232–6. 10.1523/JNEUROSCI.2695-04.2004 15385606 PMC6729696

[157] Yang Y Wang Y Li S Xu Z Li H Ma L. Mutations in *SCN9A*, encoding a sodium channel alpha subunit, in patients with primary erythermalgia. J Med Genet. (2004) 41:171–4. 10.1136/jmg.2003.012153 14985375 PMC1735695

[158] Doty CN. *SCN9A*: another sodium channel excited to play a role in human epilepsies. Clin Genet. (2010) 77:326–8. 10.1111/j.1399-0004.2009.01366_1.x 20095983

[159] Zhang T Chen M Zhu A Zhang X Fang T. Novel mutation of *SCN9A* gene causing generalized epilepsy with febrile seizures plus in a Chinese family. Neurol Sci. (2020) 2020:1–5. 10.1007/s10072-020-04284-x 32062735 PMC7359139

[160] Kasperaviciute D Catarino C Matarin M Leu C Novy J Tostevin A. Epilepsy, hippocampal sclerosis and febrile seizures linked by common genetic variation around *SCN1A*. Brain. (2013)136:3140–50. 10.1093/brain/awt233 24014518 PMC3784283

[161] EPICURE Consortium, EMINet Consortium Steffens M Leu C Ruppert A Zara F. Genome-wide association analysis of genetic generalized epilepsies implicates susceptibility loci at 1q43, 2p16.1, 2q22.3 and 17q21.32. Hum Mol Genet. (2012) 21:5359–72. 10.1093/hmg/dds373 22949513

[162] International League Against Epilepsy Consortium on Complex Epilepsies. Genetic determinants of common epilepsies: a meta-analysis of genome-wide association studies. Lancet Neurol. (2014) 13:893–903. 10.1016/S1474-4422(14)70171-1 25087078 PMC4189926

[163] Feenstra B Pasternak B Geller F Carstensen L Wang T Huang F. Common variants associated with general and MMR vaccine-related febrile seizures. Nat Genet. (2014) 46:1274–82. 10.1038/ng.3129 25344690 PMC4244308

[164] International League Against Epilepsy Consortium on Complex Epilepsies. Genome-wide mega-analysis identifies 16 loci and highlights diverse biological mechanisms in the common epilepsies. Nat Commun. (2018) 9:5269. 10.1038/s41467-018-07524-z 30531953 PMC6288131

[165] Kasperaviciute D Catarino C Heinzen E Depondt C Cavalleri G Caboclo L. Common genetic variation and susceptibility to partial epilepsies: a genome-wide association study. Brain. (2010) 133:2136–47. 10.1093/brain/awq130 20522523 PMC2892941

[166] Wijmenga C Zhernakova A. The importance of cohort studies in the post-GWAS era. Nat Genet. (2018) 50:322–8 10.1038/s41588-018-0066-3 29511284

[167] Esterhuizen AI Carvill GL Ramesar RS Kariuki SM Newton C Poduri A. Clinical application of epilepsy genetics in africa: is now the time? Front Neurol. (2018) 9:1–7. 10.3389/fneur.2018.00276 29770117 PMC5940732

[168] Fodjo S Nelson J Remme JH Preux PM Colebunders R. Meta-analysis of epilepsy prevalence in West Africa and its relationship with onchocerciasis endemicity and control. Int Health. (2020) 12:192–202. 10.1093/inthealth/ihaa012 32141502 PMC7320426

[169] Akinsulore A Adewuya A. Psychosocial aspects of epilepsy in Nigeria: a review. African J Psychiatry. (2010) 13:351–6. 10.4314/ajpsy.v13i5.63100 21390405

[170] Mukuku O Nawej P Bugeme M Nduu F Mawaw PM Luboya ON. Epidemiology of epilepsy in Lubumbashi, Democratic Republic of Congo. Neurol Res Int. (2020) 2020:5621461. 10.1155/2020/5621461 32411462 PMC7204195

[171] Ba-Diop A Marin B Druet-Cabanac M Ngoungou EB Newton CR Preux PM. Epidemiology, causes, and treatment of epilepsy in sub-Saharan Africa. Lancet Neurol. (2014) 13:1029–44. 10.1016/S1474-4422(14)70114-0 25231525 PMC5497080

[172] Owolabi LF Adamu B Jibo AM Owolabi SD Imam AI Alhaji ID. Neurocysticercosis in people with epilepsy in Sub-Saharan Africa: a systematic review and meta-analysis of the prevalence and strength of association. Seizure Eur J Epilepsy. (2020) 76:1–11. 10.1016/j.seizure.2020.01.005 31935478

